# Novel Baicalein-Derived Inhibitors of *Plasmodium falciparum*

**DOI:** 10.3390/pathogens12101242

**Published:** 2023-10-13

**Authors:** Chandra Sekhar Gudla, Vignesh Selvam, Siva Shanmugam Selvaraj, Renu Tripathi, Prince Joshi, Salique Hassan Shaham, Mayas Singh, Radha Krishan Shandil, Saman Habib, Shridhar Narayanan

**Affiliations:** 1 Foundation for Neglected Disease Research, Bangalore 561203, Karnataka, India; 2 Molecular Microbiology and Immunology, CSIR—Central Drug Research Institute, Lucknow 226301, Uttar Pradesh, India; 3 Biochemistry and Structural Biology, CSIR—Central Drug Research Institute, Lucknow 226301, Uttar Pradesh, India

**Keywords:** malaria, *Plasmodium falciparum*, *Plasmodium yoelii* N67, baicalein, FNDR-10132

## Abstract

Malaria, a life-threatening mosquito-borne disease caused by *Plasmodium* parasites, continues to pose a significant global health burden. Despite notable progress in combating the disease in recent years, malaria remains prevalent in many regions, particularly in Southeast Asia and most of sub-Saharan Africa, where it claims hundreds of thousands of lives annually. Flavonoids, such as the baicalein class of compounds, are known to have antimalarial properties. In this study, we rationally designed and synthesized a series of baicalein derivatives and identified a lead compound, FNDR-10132, that displayed potent in vitro antimalarial activity against *Plasmodium falciparum* (*P. falciparum*), both chloroquine-sensitive (60 nM) and chloroquine-resistant (177 nM) parasites. FNDR-10132 was evaluated for its antimalarial activity in vivo against the chloroquine-resistant strain *Plasmodium yoelii* N67 in Swiss mice. The oral administration of 100 mg/kg of FNDR-10132 showed 44% parasite suppression on day 4, with a mean survival time of 13.5 ± 2.3 days vs. 8.4 ± 2.3 days of control. Also, FNDR-10132 displayed equivalent activity against the resistant strains of *P. falciparum* in the 200–300 nM range. This study offers a novel series of antimalarial compounds that could be developed into potent drugs against chloroquine-resistant malarial parasites through further chemistry and DMPK optimization.

## 1. Introduction

Baicalein derivatives have emerged as promising candidates for combating malaria [1], a widespread and severe infectious disease caused by *Plasmodium* parasites [2]. In 2021, approximately 247 million malaria cases were reported worldwide, with the majority concentrated in Africa. The COVID-19 pandemic caused disruptions in access to treatment between 2019 and 2021, resulting in an estimated 13.4 million additional cases [3]. Nigeria, the Democratic Republic of the Congo, Uganda, and Mozambique accounted for nearly half of all global malaria cases, and the African region alone accounted for approximately 95% of cases, with an estimated 234 million cases in 2021.

However, the malleability exhibited by mosquitoes and the Plasmodium parasite has resulted in a progressive augmentation of resistance to pharmaceutical drugs and insecticides. Notably, cases of resistance to artemisinin-based combination therapies (ACTs) have been reported in specific countries. However, the global spread of these resistant strains highlights a significant risk with severe consequences and a catastrophic impact. In Africa, resistance to multiple insecticides has been detected in approximately two-thirds of endemic regions where malaria is prevalent. Moreover, about 80% of infections exhibit no symptoms, while Plasmodium vivax parasites persist in a dormant state for extended periods from months to years after the initial infection [4]. The development of drug resistance in Plasmodium parasites has posed challenges to the effectiveness of available antimalarial drugs, including pyrimethamine [5], proguanil [6], chloroquine [7], mefloquine [8], atovaquone [9], quinine [10], and artemisinin [11]. Thus, there is an immediate need for new and innovative drugs to combat malaria. A scoping review by Abd-Rahman and colleagues identified 31 antimalarial candidates under development, 27 candidates in phase I, 20 in phase II, and combined phases I and II studies for 3 candidates. Of these, twenty-four studies have published results, and the remaining twenty-six are yet to be published. For the unpublished studies, recruitment was completed for twelve studies, six were in the recruitment process, three were terminated, two were withdrawn, two were yet to start recruitment, and one study status was unknown [12].

Recently, there has been a renewed interest in natural product-based drug discovery. Flavonoids, a group of polyphenolic natural compounds found in tea, vegetables, red wine, and fruits, have garnered substantial attention due to their extensive research-backed health benefits [13] and their anti-inflammatory [14], anti-cancer [13], and anti-thrombogenic properties [15]. Furthermore, flavonoids exhibit anti-microbial effects against infectious agents such as dengue and chikungunya viruses, Leishmania, and Trypanosoma [16] while also holding therapeutic potential in osteoporosis. This prompted us to explore flavonoids, including baicalein derivatives, as potential sources for developing novel antimalarial drugs.

The exploration of baicalein derivatives as potential antimalarial agents offers several advantages. Firstly, baicalein is derived from natural sources, which adds to its appeal in drug development [15]. Secondly, by introducing structural modifications to baicalein derivatives, their bioavailability, pharmacokinetics, and overall antimalarial efficacy can be improved [17]. Baicalein is a main ingredient of a traditional medicine in China, Rhizoma Huang Qin (*Scutellaria altissima* L.), and it has demonstrated therapeutic effects against Parkinson’s and Alzheimer’s diseases [18]. Furthermore, derivatives of baicalein, such as 3,7-dihydroxy-3′-(4-hydroxy-3-methylbutyl)-5,6,4′-trimethoxyflavone,5,7-dihydroxy-3′-(2-hydroxy-3-methyl-3-butenyl)-3,6,4′-trimethoxy flavone and 3,7-dihydroxy-3′-(2-hydroxy-3-methyl-3-butenyl)-5,6,4′-trimethoxyflavone, have shown potent antimalarial activity against *P. falciparum* strains, although their cytotoxicity is still unknown [19]. Extracts derived from *Andira inermis* leaves, containing astilbin, afrormosin, calycosin, engelitin, formononetin, prunetin, and 3′-O-trans-cinnamoyl-astilbin, have also demonstrated significant activity against *P. falciparum* strains, making them promising candidates for malaria treatment [20]. Thus, incorporating baicalein derivatives and other flavonoids into various therapeutic applications presents an opportunity to explore and harness their immense medicinal potential [21].

In the present study, we have synthesized novel derivatives of baicalein and evaluated their potential as antimalarial agents. By exploring the structure–activity relationships of baicalein derivatives, we could optimize their pharmacological properties, enhance antimalarial activity, and reduce cytotoxicity. This research contributes to the ongoing efforts in antimalarial drug discovery and holds promise for developing new and improved treatments against malaria.

## 2. Materials and Methods

### 2.1. Synthesis of Baicalein Derivatives

The chemical synthesis of the baicalein derivatives generated in this study is described in detail under Supplemental Material.

**Scheme 1:**
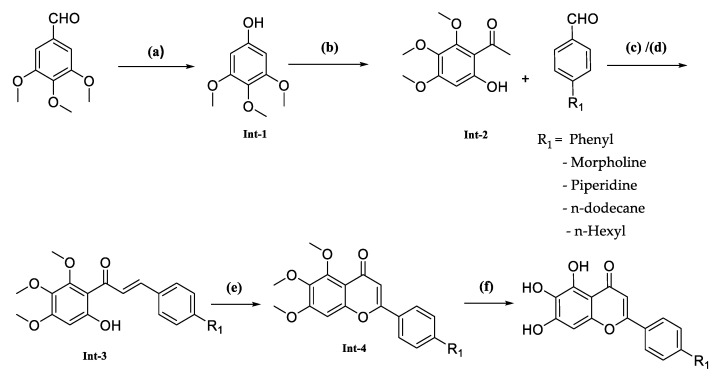
Reagents and conditions: (**a**) (i) m-CPBA, Sodium bicarbonate, DCM, RT, 16 h. (ii) K_2_CO_3_, methanol; (**b**) BF_3_.Et_2_O, acetic acid, 90 °C, 3 h. (**c**) Potassium Hydroxide, Ethanol, RT, 12 h; (**d**) Lithium diisopropylamide (LDA), THF, −78 °C to RT, 16 h (**e**) Iodine, DMSO, 100 °C, 16 h; and (**f**) Acetic acid, 47% Hydrobromic acid, 120 °C, 48 h. Int—Intermediate.

The synthesis of this series of compounds involved multiple steps as described in Scheme 1, starting with converting commercially available 3,4,5-trimethoxybenzaldehyde into 3,4,5-trimethoxyphenol. This conversion was achieved by reacting 3,4,5-trimethoxybenzaldehyde with m-CPBA. Next, an acetyl functional group was introduced to the phenyl ring using a Lewis acid, such as BF_3_·Et_2_O, which formed the desired intermediate-2. Following this, two different methods were employed to obtain the condensed product, intermediate-3. In step (c), intermediate-2 and an aldehyde condensation were combined using a strong inorganic base such as KOH in ethanol. Notably, in step (d), the compound FNDR-10142 was synthesized by reacting commercially available dodecanal with tri-methoxy substituted 2-hydroxy acetophenone in the presence of lithium diisopropylamide (LDA) in tetrahydrofuran (THF). Subsequently, these intermediates were cyclized with iodine using a catalytic amount of iodine, followed by demethylation with HBr in acetic acid to produce the desired compounds (Table 1).

**Scheme 2:**
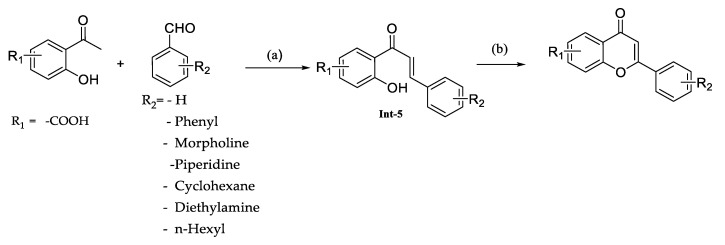
Reagents and conditions: (**a**) Potassium Hydroxide, Ethanol, RT, 12 h; (**b**) Iodine, DMSO, 100 °C, 16 h.

The reaction involved the condensation of substituted acetophenones with aldehydes using an inorganic base such as KOH in ethanol and resulted in the formation of intermediate-5. Subsequently, these intermediates underwent cyclization with iodine as a catalyst to produce the desired compounds (Scheme 2,Table 2).

**Scheme 3:**
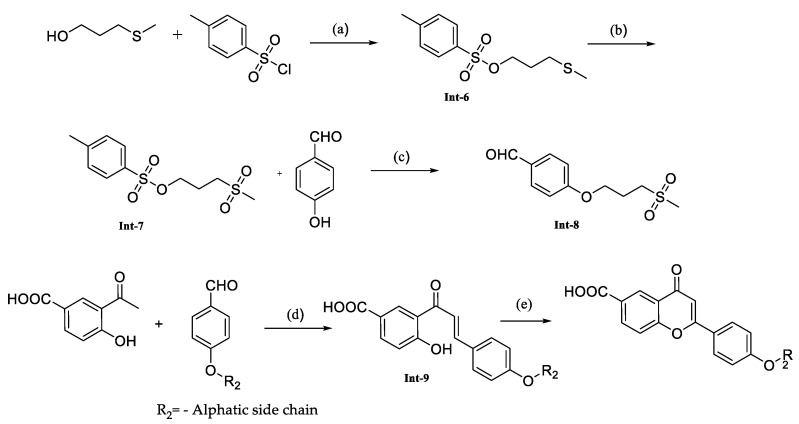
Reagents and conditions: (**a**) Dichloromethane, Pyridine, RT, 12 h; (**b**) Oxone, Water, Methanol, RT, 12 h; (**c**) K_2_CO_3_, DMF, 100 °C, 12 h; (**d**) Potassium Hydroxide, Ethanol, RT, 12 h; and (**e**) Iodine, DMSO, 100 °C, 16 h.

The synthesis of the compounds involved a stepwise process using commercially available substituted benzaldehydes by following the process described in Scheme 2. The compound FNDR-11096 synthesized by following the Scheme 3. Initially the alcohol was converted to a sulfonic ester by reacting it with Tosyl chloride, resulting in intermediate-6. Subsequently, the thioether moiety was oxidized to sulfone as intermediate-7. The next step involved the reaction of intermediate-7 with benzaldehyde using potassium carbonate as a base, which formed intermediate-8. In the condensation step, strong inorganic bases, specifically KOH in ethanol, were employed to facilitate the formation of various intermediate compounds. These intermediates were then subjected to cyclization, following a similar approach as described in Scheme 1, in the presence of catalytic iodine. This final cyclization step formed the desired compounds (Table 3).

**Scheme 4:**
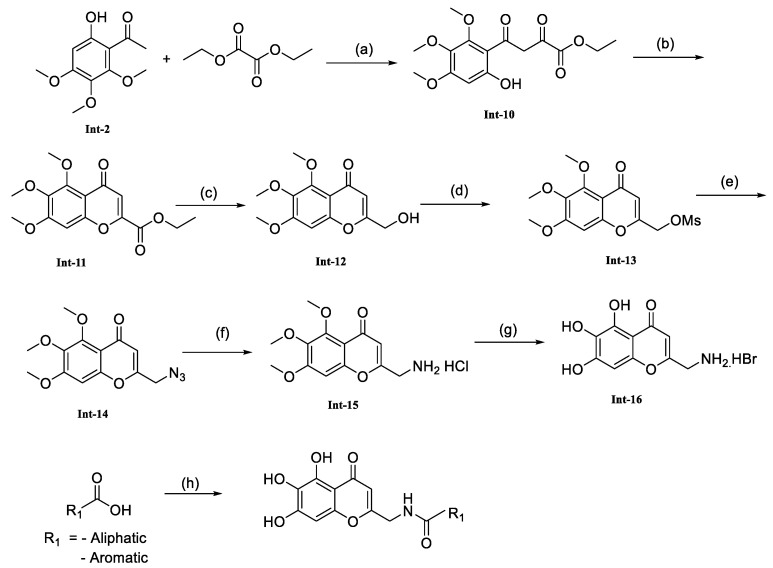
Reagents and conditions: (**a**) Sodium metal, Ethanol, diethyl oxalate, 80 °C, 16 h; (**b**) Ethanol, conc. HCl, 80 °C, 16 h; (**c**)THF, Ethanol, 0 °C, Sodium borohydride, 0–5 °C, 3 h; (**d**) Dichloromethane, Triethylamine, Methane sulfonyl chloride, 0–5 °C, 3 h; (**e**) DMF, Sodium azide, RT, 2 h; (**f**) THF, Diethyl ether, Triphenylphosphine, 0 °C, 1 h, 6 N HCl, RT, 16 h; (**g**) Acetic acid, 47% Hydrobromic acid, 120 °C, 48 h; and (**h**) DMF, HATU, DIPEA, RT, 16 h.

We have synthesized the amide linker compounds with tri-hydroxy substitution by following the Scheme 4 synthetic route. This involved a reaction between intermediate-2 and diethyl oxalate to generate the dicarbonate derivative. Then, it was cyclized by refluxing with conc. HCl, resulting in the formation of Intermediate-11, an ester compound. Following this, the ester was reduced to alcohol using sodium borohydride and further converted into a mesylate by reacting it with methane sulfonyl chloride, yielding intermediate-13. Mesylated intermediate-13 was subjected to azide formation by using DMF and sodium azide. The azide compound was then reduced to an amine using THF, diethyl ether, and PPh_3_ as the reducing agents, forming an amine. Intermediate-15 underwent demethylation using 47% hydrobromic acid to remove the methyl group. Subsequently, amide formation was achieved by coupling with commercially available carboxylic acids using HATU and DIPEA as coupling agents, which produced the desired compounds (Table 4).

^1^H and ^13^C NMR spectra confirmed the structures of all synthesized final products. The mass and purity of the compounds were determined by liquid chromatography–mass spectrometry (LC-MS), confirming a purity of at least 95%.

### 2.2. Microsomal Stability Assay

Dipotassium hydrogen phosphate, Potassium dihydrogen phosphate, and formic acid were obtained from Merck, Rahway, NJ, USA. Methanol–acetonitrile was procured from Biosolve Chimie, Dieuze, France. Mouse liver microsomes, 20 mg/mL, were received from a third-party vendor in India. Imipramine HCl (catalog no. I0971) and Carbamazepine (catalog no. O0363) were procured from TCI, Gurugram, India. ß-NADPH (product code-99197) was procured from Sisco Research Laboratories, Mumbai, India.

The in vitro metabolic stability was determined in mouse liver microsomes [22]. To prepare a mouse liver microsomes (MLM) homogenate, 100 µL of microsomes was resuspended in 100 mM phosphate buffer (0.607 g K_2_HPO_4_ and 0.212 g KH_2_PO4 dissolved in 50 mL of de-ionized water), achieving a working concentration of 0.625 mg/mL. Positive control (Imipramine) and test compounds (FNDR-10132, 10136, 10139, and 10999) were spiked individually into the MLM homogenate at a final concentration of 1 µm. To pre-incubate, they were placed in a water bath at 37 °C for 5 min. At least 30 µL was collected in triplicates (0 min samples), and the reaction was quenched using 90 µL of acetonitrile, which contained 500 ng/mL of Carbamazepine as an internal standard. Nicotinamide Adenine Dinucleotide Phosphate (NADPH) in phosphate buffer was added to the remaining MLM at a 1 mM final concentration and further incubated at 37 °C. At the indicated time intervals, 30 µL aliquots were sampled in triplicates, and the reaction was quenched with acetonitrile containing Carbamazepine as the internal standard [23]. The samples were centrifugated for 10 min at 13,000 rpm, and the supernatant was separated for further analysis.

### 2.3. Plasma Protein Binding Assay

Plasma protein binding was performed following the RED method, i.e., rapid equilibrium dialysis [24,25]. The test compound was prepared to achieve a 5 µM final concentration when spiked in blank plasma. 200 µL of the spiked sample was aliquoted into the sample chamber of RED inserts. A total of 350 µL of the buffer was added into another chamber of the inserts. The plate was covered and incubated at 37 °C in an orbital shaker at 100 rpm for at least 4 h. Aliquots of 100 µL were taken from the buffer and plasma chambers into another tube. A total of 100 µL of plasma was added to the buffer sample, and 100 µL of buffer was added to the plasma sample. We added 400 µL of acetonitrile containing the internal standard to each tube. It was vortexed for 2 min and centrifuged at 13,000 rpm for 10 min at 4 °C. Then, the supernatant was placed into the sample vials and loaded for LC-MS/MS analysis.

The analytical method for microsomal stability and protein binding assay is as follows: Chromatographic elution was achieved following isocratic flow on an X-Terra MS C18 50 × 4.6 mm, 2.5 µm, Waters, USA, maintained at 40 °C. The mobile phase contained aqueous 0.1% formic acid in water in solvent A and acetonitrile acidified with 0.1% formic acid in solvent B delivered at 0.300 mL/min. The flow was 15% from solvent A and 85% from solvent B for 4.5 min. A total of 7 μL of the sample was injected for the assay. Carbamazepine was used as an internal standard as it was observed to be compatible with the extraction method and test compounds. Detection was performed through multiple reaction monitoring (MRM). The ion transitions are as follows: Imipramine, FNDR-10132, FNDR-10136, FNDR-10139, FNDR-10999, and IS at *m*/*z* 281.1→86.0, 355.2→115.1, 354.0→298.0, 350.0→186.1, 349→305.2, and *m*/*z* 237.0→194.0, respectively. An optimized cone voltage and collision energy were used to ionize the compounds. The MS parameters follow as follows: cone gas flow 50 (L/h); ion source temperature 150 °C; desolvation gas flow 900 (L/h); desolvation temperature 400 °C; and collision gas flow 0.10 (mL/min).

### 2.4. Calibration Standards and Quality Control Samples

A stock solution of FNDR-10132 was produced by dissolving in DMSO to achieve a 2 mg/mL concentration. The intermediate stock dilutions were prepared with 0.1% formic acid in 20% acetonitrile in water as a diluent.

The internal standard’s stock solution (1 mg/mL) was prepared by dissolving Carbamazepine in DMSO, and its working solution was prepared by further diluting the stock solution using 0.1% formic acid in acetonitrile as a diluent to achieve 100 ng/mL as a final concentration.

### 2.5. Sample Preparation

Sample preparation involved a simple protein precipitation technique determining FNDR-10132 in the mice plasma. A 5% spiking protocol was followed to prepare STDs and QCs using intermediate stock dilutions. A total of 20 µL of spiked samples from STDs and QCs and 20 µL from subject samples were aliquoted into another tube. A total of 180 µL of the IS working solution was added to this tube and vortexed for 2 min. These samples were centrifugated at 4 °C for 10 min at 13,000 rpm, and the supernatant was transferred to autosampler vials and analyzed with the LC-MS/MS instrument.

### 2.6. LC-MS/MS Analysis

The sample analysis was performed using UPLC-MS/MS, i.e., Waters TQD mass spectrometry coupled with UPLC. The system operated with ESI positive ion mode, and the data interpretation was achieved using Target Lynx™.

Chromatographic elution was achieved by isocratic elution on a Zorbax SB-C18, 2.1 × 50 mm, Agilent, Santa Clara, CA, USA, maintained at 40 °C. The mobile phase combines solvents A and B with 0.1% formic acid in a 5 mM ammonium acetate buffer and 0.1% formic acid in acetonitrile, respectively. Isocratic flow at 90% from solvent B was delivered at 0.350 mL/min. The analysis was performed by using 7 μL of the sample. Carbamazepine was used as an internal standard as it was observed to be compatible with the extraction method along with FNDR-10132. Detection was achieved through multiple reaction monitoring (MRM) with transitions of FNDR-10132 and IS at *m*/*z* 355.2→115.1 and 237.0→194.0, respectively. A desired cone voltage and collision energy were used to ionize the compound. Optimized MS parameters were as follows: ion source temperature 150 °C; collision gas flow 0.10 (mL/min); cone gas flow 50 (L/h); desolvation temperature 400 °C; and desolvation gas flow 900 (L/h).

### 2.7. In Vitro

#### 2.7.1. Parasite Strains

Parasite lines used in the study were *P. falciparum* 3D7 (*Pf*3D7 Chloroquine-sensitive) and *Plasmodium falciparum* K1 (*Pf*K1 Chloroquine-resistant) sourced from the Malaria Research and Reference Reagent Resource Center (MR4), ATCC, Manassas, VA, USA. FNDR-10132 was tested against a cross-resistance panel at Swiss TPH, Allschwil, Switzerland.

#### 2.7.2. Parasite Culture

As per the modified protocol stated by Trager and Jensen, 1976, *Plasmodium falciparum* parasite lines (*Pf*3D7 and *Pf*K1) were cultured. *Plasmodium* strains were maintained in RPMI-1640 medium at 3–5% hematocrit supplemented with 0.2% *w*/*v* glucose, 0.5% *w*/*v* AlbuMaxII, 0.2% *w*/*v* NaHCO_3_, and 30 μg/mL gentamycin, and, additionally, 15 μM hypoxanthine was added and incubated at 37 °C with 5% O_2_, 5% CO_2_, and 90% N_2_. After 24 h, the medium was changed. After attaining high parasitemia (6–8% mature stage parasite), the culture was sub-passaged with fresh human RBCs.

#### 2.7.3. Preparation of Blood Smear

The parasite maturity and parasitemia were assessed by making thin blood films of *Plasmodium* culture. To make a thin blood film, 2–5 μL of RBCs from the culture was taken and smeared on the glass slide, air-dried, fixed with 100% methanol, and stained for 30 min using a 30% Giemsa in staining buffer solution. Slides were evaluated microscopically at 1000× magnification using an oil immersion lens. Parasitemia was measured by collecting and counting the number of infected and uninfected red blood cells up to 10,000.

#### 2.7.4. Preparation of Human RBCs for Culture

Human blood was collected aseptically in an ACD (Acid Citrate Dextrose) solution with the approval of the institutional human ethics committee (CDRI/IEC/2019/A8). The blood was centrifuged at 2000 rpm for 10 min; leukocyte aggregates and residual plasma were removed by aspiration and then used in the parasite culture. The remaining erythrocyte pellet was washed thrice with complete RPMI (CRPMI) at 2000 rpm for 10 min to remove white blood cells and then suspended to 50% hematocrit in CRPMI. The cells were stored at 4 °C and could be kept for 15 days.

#### 2.7.5. Parasite Synchronization (D-Sorbitol Synchronization)

A 5% *w*/*v* D-sorbitol method was used for parasite synchronization. Briefly, 5% of D-sorbitol (Sigma Aldrich, St. Louis, MO, USA) was prepared in MilliQ and passed through a 0.22 µM syringe filter. The parasite supernatant was removed after centrifugation at 2000 rpm for 5 min. Then, a 1:5 ratio of D-sorbitol (1 part parasite pellet and 5 part d-sorbitol) was added to the parasite palette and incubated at 37 °C for 15 min. Then, the tube was centrifuged at 2000 rpm for 5 min, and the resulting pellet was washed thrice with RPMI to remove late-stage parasites by lysis caused by D-sorbitol and then suspended in CRPMI.

#### 2.7.6. Preparation of Stock Solutions for the Compounds

All tested compounds were dissolved in a DMSO solvent and a prepared stock solution of 10 mM. The reference drug, Chloroquine diphosphate, was dissolved in CRPMI. Working solutions were made from stock solution after diluting in a CRPMI medium during the experiment. The maximum concentration of DMSO used in this study was <1%. It had no parasiticidal effect.

#### 2.7.7. Antimalarial Assay (IC_50_ Determination)

The *P. falciparum* (0.8–1% parasitemia and 1% hematocrit) synchronous culture was exposed for 72 h (at 37 °C, 5% O_2_, 5% CO_2_, and 90% N_2_) to the serially diluted compound in 96-well plates. A total of 100 µL of RBCs lytic buffer (5 mM EDTA, 20 mM Tris pH 7.5, 0.08% Triton X-100, and 0.008% Saponin) containing the SYBR green 1-X final concentration was distributed to each well and incubated for one hour at room temp in the dark. The plates were read under a fluorescence reader at an excitation of 485 ± 20 nm and emission of 535 ± 25 nm. The IC50 values were calculated based on the DNA content of the parasite relative to its control [26]. An inhibitory concentration of 50% (IC50) was determined using the MS-EXEL template. The signal-to-noise ratio was found to be 1:5–10.

#### 2.7.8. Cytotoxicity Assay against Vero Cell Line

Vero cells were initially washed with PBS and trypsinized, and a 1 × 10^5^ cells/mL suspension was prepared. A total of 100 µL of a cell suspension was distributed to each well of the 96-well microtiter plates and allowed to adhere overnight. The next day, serial dilutions of selected test compounds were made in the plates and incubated for 72 h. After 72 h, a ten-microliter resazurin solution (12.5 mg/100 mL PBS) was added to each well. After 3 h, plates were read using a fluorescence reader (Biotek) at an excitation wavelength of 530 nm and an emission wavelength of 590 nm (Sperandeo and Brun, 2003). The MS-EXEL template determined the cytotoxic concentration (CC50) for selected compounds.

The selectivity indices of the compounds were determined to select them for in vivo evaluation using the following formula:

$$Selectiveindex=\frac{Mediancytotoxicityconcentration({CC}_{50})}{MedianInhibitoryconcentration({IC}_{50})}$$

#### 2.7.9. *P. falciparum* Male/Female Gamete Formation Assay (DGF ASSAY)

The FNDR-10132 was evaluated in a male and female gametocyte functional viability assay by following the procedure described in Ruecker et al. [26].

### 2.8. Animal Studies

#### 2.8.1. Experimental Animals and Parasite

All animal experiments were performed following the institutional animal ethics committee (IAEC). Laboratory-bred Swiss albino mice (both male and female) weighing 20–22 g were obtained from breeding colonies at the National Laboratory Animal Centre at the CSIR-CDRI, Lucknow, with due permission from the animal ethics committee (IAEC/2018/16/Renew-1/Dated-05/04/2019). The rodent malaria parasite *Plasmodium yoelii* N67 (chloroquine-resistant) was used for the study. This parasite was maintained by serial passage in healthy Swiss mice of either sex.

#### 2.8.2. In Vivo Antimalarial Assay

Swiss mice were inoculated with a standard inoculum of 1 × 106 *P. yoelii* N67 parasitized erythrocytes intraperitoneally. The 22-gauge hypodermic needle was used to infect the mice and was injected with a 0.5 mL (infected RBCs) volume. Treatment was initiated from day 0 to day 3 (total 4 days) once daily, and different routes of administration, viz., oral, intravenous, intraperitoneally, and subcutaneous, were used. For in vivo evaluation, compounds were dissolved in 300 µL of DMSO and were made up with MilliQ. The efficacy of in vitro active compounds was evaluated for in vivo activity at different doses, and the desired daily dose was administered in a 0.2 mL volume. A drop of blood from the mice’s tails was collected regularly throughout the experiment for parasitemia estimation. The group’s mean value was calculated using the percentage suppression of parasitemia compared with the untreated control group.

#### 2.8.3. In Vivo Study Design

Healthy BALB/c mice weighing 20 to 25 gm were maintained under standard laboratory conditions before the experiments. An accurately weighed amount of FNDR-10132 was suspended in a mixture of 0.5% HPMC and 0.1% tween-80 to prepare an oral suspension formulation, whereas for the IV formulation, the compound was dissolved in a mixture that contained 5% dimethyl acetamide, 10% solutol, and 85% normal saline. The animals were fed before the compound administration.

The compound was orally administered in BALB/c mice at 30 and 100 mg/kg and the IV route at 5 and 30 mg/kg. However, no plasma levels were observed. Hence, an oral PK at 100 mg/kg of the test compound was performed after dosing the animals with Amino benzotriazole (ABT) at 100 mg/kg. Similarly, 200 mg/kg of Probenecid was dosed before the IV dose of the test compound at 30 mg/kg. Probenecid and Amino benzo triazole were dosed 1 h before the dosage of the test compounds. Probenecid was used to delay renal clearance, and ABT was used as a Pan-Cytochrome P450 inhibitor to increase the bioavailability of FNDR-10132. Blood samples (~50 μL) were collected in a K_2_EDTA tube at exact time points. Samples were centrifuged at 4500 rpm for 10 min, after which plasma was separated and taken for sample preparation. The separated plasma samples were collected and stored at −80 °C until taken for analysis. Non-compartmental pharmacokinetic parameters were determined using a PK calculation tool.

## 3. Results

Baicalein is a natural flavonoid compound derived from the root of *Scutellaria baicalensis*, a traditional Chinese herb. Several studies have investigated the effects of baicalein against malaria, both in vitro (in laboratory settings) and in vivo (in animal models). In a study published in the journal Parasitology Research in 2012, baicalein effectively suppressed the growth of *P. falciparum*, the most prevalent parasite causing malaria in humans, in cultured human red blood cells [27]. Baicalein prominently manifests its anti-inflammatory properties through the active intervention and inhibition of producing pro-inflammatory cytokines and enzymes. By reducing inflammation, baicalein may help alleviate some of the symptoms associated with malaria [28].

Our pursuit to discover an antimalarial compound commenced with the notion that compounds designed based on baicalein may be more likely to exhibit effectiveness against malaria. As a first step, we synthesized five baicalein derivatives with a diversified class of groups. From aryl, we picked up simple phenyl (FNDR-10131), n-hexyl, and dodecane groups (FNDR-10132 and FNDR-10142) from aliphatic and morpholine and piperidine from aliphatic heterocyclic compounds (FNDR-10133 and FNDR-10136). These compounds were synthesized by following the procedure described in Scheme 1.

The antimalarial activity of these compounds was measured by targeting the asexual blood-stage parasite *P. falciparum* (chloroquine-sensitive strain 3D7). Of the five compounds tested, all showed activity with IC50 values of 0.06–1.2 μM (Table 1, Table 2, Table 3 and Table 4), while baicalein’s antimalarial activity was 32 µM. They displayed a significant increase in antimalarial activity by extending the baicalein scaffold further. There was no difference in the antimalarial activity of aryl, aliphatic, or aliphatic heterocycles among the lipophilic groups, which all showed identical antimalarial activity. However, morpholine showed a 4–5-fold reduction in potency, indicating a hydrophobicity preference.

In parallel, we synthesized another set of compounds with carboxylic acid functional groups at different positions to replace the trihydroxy group on the chromone ring. About ten compounds were produced with the carboxylic acid group at the sixth position on the chromone ring. However, these compounds were not very potent, and the activity was significantly reduced. The simple baicalein mimics with the carboxylic acid group, FNDR-10130, showed no activity at the 25 µM tested concentration. The side chains found to be highly active with trihydroxy groups were rendered inactive by the carboxylic acid group on the chromone ring. The compounds FNDR-10137, FNDR-10138, FNDR-10139, and FNDR-11009 exhibited antimalarial activity against asexual blood-stage parasites with IC50 values of 13.16 µM, 9.65 µM, 4.06 µM, and 10.36 µM, respectively. Replacement of the trihydroxy group on the chromone ring with a carboxylic acid group significantly reduced antimalarial activity.

We synthesized the compounds FNDR-10999, which has a fragment of cyclohexane, and FNDR-11001, which has N, N-di ethyl substituents. Both compounds displayed similar IC50 values around 10 µM in the asexual blood-stage assay and did not lead to improvement in the potency.

Later, we produced three compounds (FNDR-11000, FNDR-11011, and FNDR-11096) with aliphatic chains of various lengths introduced to the scaffold’s phenolic group. In order to increase the permeability and optimize the physicochemical properties, the molecule FNDR-11096 was synthesized with a sulfone group. However, none of the compounds were active when the carboxylic acid group was used as an alternative to trihydroxy substitutions.

Additionally, in order to better understand the significance of substitution position, a series of four compounds (FNDR-11003, FNDR-11012, FNDR-11013, and FNDR-11014) with a carboxylic acid group at the seventh position were created. The compounds’ antimalarial activities were unaffected by the change in position, and they remained inactive.

We explored another new series (compounds FNDR-10143, FNDR-10146, FNDR-10148, FNDR-10149, and FNDR-10150) where the 2-aminomethyl group was introduced at the second position, and the derivatives were generated via amide bond linkage (Results, Table 4). The linker length of carboxylic acids was examined to optimize the suitable linker length and effect on the antimalarial activity. None of these compounds demonstrated better potency than trihydroxy series compounds (Table 4). However, they showed good activity compared to the carboxylic acid series (Table 2). FNDR-10148 exhibited the highest potency from this series with an IC50 of 0.9 µM. Therefore, we selected the compounds from other series and evaluated them for their PK and metabolic stability properties.

**Figure 1:**
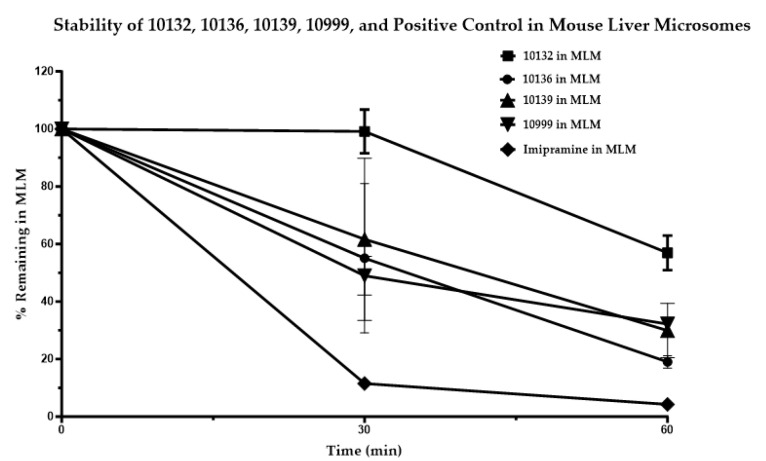
Stability of the test compounds in the mouse liver microsomes with Imipramine as a positive control.

The stability of the positive control and four test compounds, FNDR-10132, FNDR-10136, FNDR-10139, and FNDR-10999, was assessed in mouse liver microsomes in the presence of ß-NADPH for 60 min at 37 °C and found to be around 56.95 ± 3.49%, 19.05 ± 1.26%, 29.92 ± 5.46%, and 32.19 ± 0.29%, respectively (Figure 1). While FNDR-10132 showed moderate stability, the other three compounds showed poor stability in mouse liver microsomes.

FNDR-10132 was found to have decent metabolic stability and good in vitro activity against *P. falciparum*. FNDR-10132 was profiled further for its P. falciparum dual gamete formation assay (PfDGFA), activity against a cross-resistance panel (6 strains) including lab-derived and clinically isolated strains, plasma stability, cytotoxicity in THP1, Vero cells, and PK in mice (Table 5).

In the *P. falciparum* dual gamete formation assay (PfDGFA), FNDR-10132 displayed poor inhibition of 9.5% in female gametocytes and 1.6% in male gametocytes at a 1 µM concentration. However, FNDR-10132 demonstrated excellent antimalarial activity in the 200–300 nM range against all mutants/strains tested (Table 6). Therefore, FNDR-10132 might operate through a unique mechanism of action as it displayed antimalarial activity despite any resistance.

### 3.1. Pharmacokinetics of FNDR-10132 in BALB/c Mice Plasma

#### 3.1.1. Bioanalytical Results and Discussion

Though the test compound displayed decent stability in the mouse liver microsomal stability assay, no bioavailability was observed in plasma and blood at various doses via the oral route of administration. However, the plasma samples collected at 1 and 5 min from the IV PKs, at a 30 mg/kg dose, showed at least a 3 µg/mL concentration. No concentration was observed post the 5 min time point samples. The bioavailability could not be increased even after dosing Amino benzotriazole and Probenecid before the test compound. ABT was used as a CYP inhibitor to slow the metabolite conversion, and Probenecid was used to delay renal clearance.

#### 3.1.2. In Vivo Efficacy Study

The compound FNDR-10132 showed promising antimalarial properties in in vivo studies against the chloroquine-resistant strain *Plasmodium yoelii* N67 in Swiss mice [34]. FNDR-10132 demonstrated a significant 44% suppression of parasite growth by day 4 of treatment when administered orally at a dosage of 100 mg/kg. Moreover, treatment with FNDR-10132 increased the mean survival time of infected mice to 13.5 ± 2.3 days in contrast to the mean survival time of only 8.5 days observed in the control group of mice (Table 7).

One crucial aspect of evaluating potential malaria treatments is assessing their safety and tolerance in the host organism. In this regard, the present study closely monitored the body weights of the mice throughout the drug treatment. Importantly, no significant weight loss was observed during the course of the treatment with FNDR-10132. This lack of substantial weight loss indicates that FNDR-10132 is well-tolerated at a dosage of 100 mg/kg (Table 8), suggesting its potential as a safe and effective antimalarial agent.

The cumulative results strongly highlight FNDR-10132’s potential as a noteworthy candidate for advancing development as an antimalarial therapeutic agent, particularly in addressing the challenge of chloroquine-resistant Plasmodium strains [35].

## 4. Conclusions

We generated a library of compounds based on baicalein in a rational design approach, and iterative synthesis allowed us to identify the compound FNDR-10132, which demonstrated antimalarial activity against the chloroquine-sensitive and -resistant *P. falciparum* strains 3D7 and K1, respectively. Further, it exhibited similar potency across clinical isolates of *P. falciparum* evaluated. The n-hexyl substitution on baicalein improved its metabolic stability in mice. Further optimization of physicochemical properties and improvement of pharmacokinetic parameters are required to achieve higher potency and translation into in vivo efficacy. Future studies are required to determine the molecular mechanism behind the antimalarial activity of FNDR-10132 and establish the structure–activity relationship with optimized PK followed by in vivo efficacy in animal models. Nevertheless, the chemical synthesis employed in this study yielded a novel chemical scaffold with antimalarial activity and opened avenues for synthesizing more potent baicalein analogs.

## Figures and Tables

**Table 1:** Antimalarial activity of Baicalein derivatives with trihydroxy groups against *Plasmodium falciparum* 3D7.

	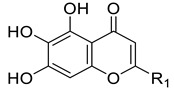	
Compound	R_1_	IC_50_ (µM)
FNDR-10131	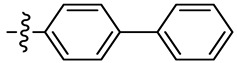	0.22
FNDR-10132	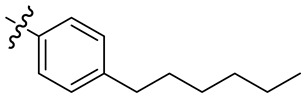	0.06
FNDR-10133	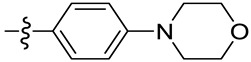	1.29
FNDR-10136	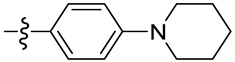	0.10
FNDR-10142	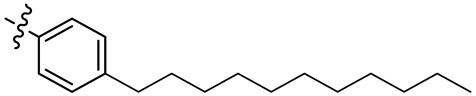	0.40

**Table 2:** Antimalarial activity of Baicalein derivatives with carboxylic acid group against *Plasmodium falciparum* 3D7.

	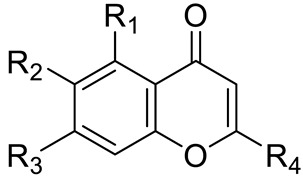				
Compound	R_1_	R_2_	R_3_	R_4_	IC_50_ (µM)
FNDR-10130	H	COOH	H	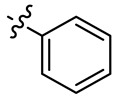	>25
FNDR-10137	H	COOH	H	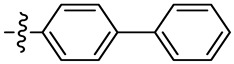	13.16
FNDR-10138	H	COOH	H	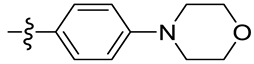	9.65
FNDR-10139	H	COOH	H	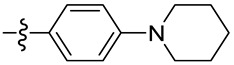	4.06
FNDR-10999	H	COOH	H	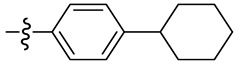	10.14
FNDR-11001	H	COOH	H	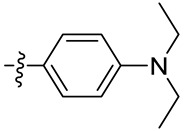	10.24
FNDR-11009	H	COOH	H	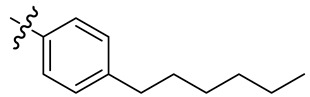	10.36
FNDR-11003	H	H	COOH	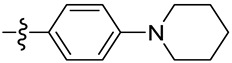	>25
FNDR-11012	H	H	COOH	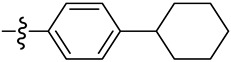	>25
FNDR-11014	H	H	COOH	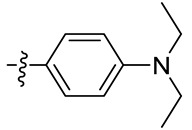	>25

**Table 3:** Antimalarial activity of Baicalein derivatives with carboxylic acid group and oxygen linkers against *Plasmodium falciparum* 3D7.

	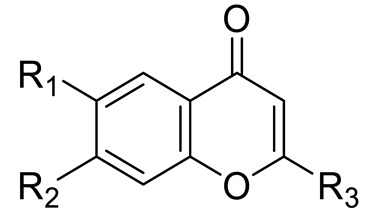			
Compound	R_1_	R_2_	R_3_	IC_50_ (µM)
FNDR-11000	COOH	H	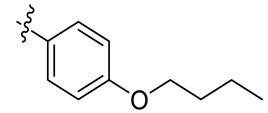	>25
FNDR-11011	COOH	H	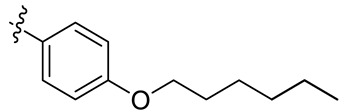	>25
FNDR-11096	COOH	H	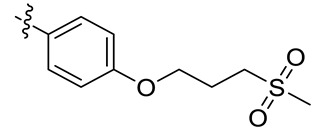	>25
FNDR-11013	H	COOH	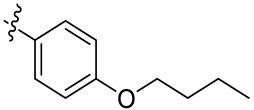	>25

**Table 4:** Antimalarial activity of Baicalein derivatives with trihydroxy with amide linker against *Plasmodium falciparum* 3D7.

	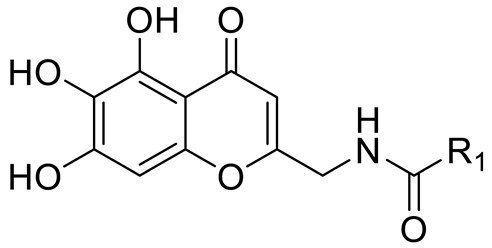	
Compound	R_1_	IC_50_ (µM)
FNDR-10143	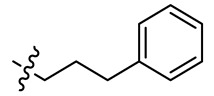	2.48
FNDR-10146	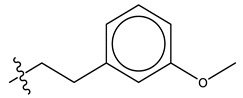	2.93
FNDR-10148	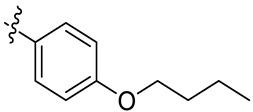	0.92
FNDR-10149	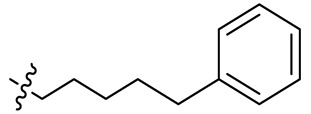	2.74
FNDR-10150	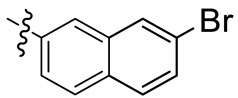	1.12

**Table 5:** The compound FNDR-10132 progression profile.

S. No.	Compound Progression Parameters	FNDR-10132
1	Antimalarial activity	
	ABS Assay	Pf (3D7): 60 nM
		Pf (K1): 177 nM
	DGF Assay—Inhibition	9.5% in female and 1.6% in male at 1 µM
2	Cross-Resistant Profile/Hypersensitivity Profile (Minimum 6 Strains Including Lab-Derived and Clinically Isolated)	Depicted in Table 6
3	Microsomal Stability (Mouse)	99% at 30 min and 57% at 60 min
4	Plasma Stability	87.61% at 30 min 80.97% at 45 min
5	Cytotoxicity	
	THP-1	90 µM
	Vero Cell	174 µM

**Table 6:** Compound FNDR-10132 cross-resistant profile.

			MMV 643121	MMV 390048	MMV 018912	MMV 000130	MMV 034055	
			DDD107498	MMV390048	DSM265	GNF156	ELQ300	FNDR-10132
	Mutated Locus	Mutations (Amino Acid Changes)	IC_50_ (nM)	IC_50_ (nM)	IC_50_ (nM)	IC_50_ (nM)	IC_50_ (nM)	IC_50_ (nM)
Dd2	wt	N/A	0.2574	18.46	6.227	9.125	6.141	327.9
Dd2 DDD107498	PfeEF2	Y186N [29]	1865	NT	NT	NT	NT	211.3
Dd2 390048	Pfpi4k	S743T [30]	NT	90.80	NT	NT	NT	289.8
Dd2 DSM265	Pfdhodh	Mutation not reported in [31]	NT	NT	150.4	NT	NT	260.5
Dd2 GNF156	Pfcarl	Ile1139Lys [32]	NT	NT	NT	2162	NT	291.7
Dd2 ELQ300	PfcytB	Ile22Leu [33]	NT	NT	NT	NT	59.72	238.0
			Fold shift IC_50_ rel. Dd2	Fold shift IC_50_ rel. Dd2	Fold shift IC_50_ rel. Dd2	Fold shift IC_50_ rel. Dd2	Fold shift IC_50_ rel. Dd2	Fold shift IC_50_ rel. Dd2
Dd2	wt	N/A	1.0	1.0	1.0	1.0	1.0	1.0
Dd2 DDD107498	PfeEF2	Y186N [29]	7247	NT	NT	NT	NT	0.6
Dd2 390048	Pfpi4k	S743T [30]	NT	4.9	NT	NT	NT	0.9
Dd2 DSM265	Pfdhodh	Mutation not reported in [31]	NT	NT	24	NT	NT	0.8
Dd2 GNF156	Pfcarl	Ile1139Lys [32]	NT	NT	NT	237	NT	0.9
Dd2 ELQ300	PfcytB	Ile22Leu [33]	NT	NT	NT	NT	9.7	0.7

**Table 7:** Antimalarial Activity of compounds Against *Plasmodium yoelii* N67 (Chloroquine-resistant) in Swiss mice.

Compound No.	Dose mg/kg ×4 Days	Route	No. of Mice	Day 4 % Suppression	% Parasitemia ± S.D on Day 4	% Parasitemia ± S.D on Day 7	% Parasitemia ± S.D on Day 10	% Parasitemia ± S.D on Day 14	% Parasitemia ± S.D on Day 21	% Parasitemia ± S.D on Day 28	Mean Survival Time (MST) Days ± SD
FNDR-10132	100 mg/kg	Oral	4	44	8.5 ± 1.39 (4)	26.15 ± 8.70 (4)	24.07 ± 3.69 (3)	46.5 ± 4.2 (2)	Dead		13.5 ± 2.3
Control	-	-	5	NA	15.18 ± 2.77 (5)	32.2 ± 5.40 (2)	22.4 (1)	Dead			8.4 ± 2.3

The numbers of survived mice are given in parentheses. The above-mentioned compound has moderate activity against Chloroquine-resistant parasites. The compounds were dissolved in 5% DMSO and made up in Milli-Q water.

**Table 8:** Weight gain in orally treated vs. control mice.

Compound Code	Weight of Mice at the Initiation of Treatment (Day 0)	Weight of Mice Day after the Last Dose of Treatment (Day 4)	Weight Gain
FNDR-10132 (100 mg/kg)	25 gm	27.5 gm	2.5 gm
Control	25 gm	29.9 gm	4.9 gm

## Data Availability

Data available on request.

## Supplementary Materials

The following supporting information can be downloaded at: https://www.mdpi.com/article/10.3390/pathogens12101242/s1. SM1 and Figures S1–S20: ^1^H NMR and LC-MS spectra of Compounds FNDR-10131 FNDR-10132, FNDR-10133, FNDR-10136, FNDR-10142, FNDR-10143, FNDR-10146, FNDR-10148, FNDR-10149 and FNDR-10150.
