# Supplementary material for: Novel Baicalein-Derived Inhibitors of Plasmodium falciparum
Source: Pathogens. 2023 Oct 13;12(10):1242. doi: 10.3390/pathogens12101242 (PMC10610289; doi:10.3390/pathogens12101242)
Supplement: Supplementary file 1 [file pathogens-12-01242-s001.zip › pathogens-2613245-supplementary.pdf]

pathogens

Supplemental Material &Methods SM1

Chemical synthesis of baicalein derivatives

We procured commercially available chemicals from BLD Pharma, Hyderabad, India and Sigma Aldrich, bangalore, India, and solvents were obtained from Avra, bangalore, India. These chemicals were used without further purification, as they were all of at least 95% purity.. All reactions were performed in a nitrogen atmosphere. The progress of the reaction was monitored using thin-layer chromatography with Merck TLC Silica gel 60 F254. ^{1}H NMR spectra were acquired on an Advance III HD Bruker spectrometer (Bruker Corporation, Fällanden, Switzerland). Chemical shifts (δ) were expressed in parts per million (ppm) relative to $CDCl_3$ or DMSO-d_6. which were used as the solvent, and to TMS as an internal standard. The following abbreviations are used to describe peak patterns when appropriate: s (singlet), d (doublet), t (triplet), q (quartet), qt (quintet), m (multiplet), app (apparent), and bs (broad singlet). For all final compounds, purity was determined by ultra-performance liquid chromatography with a UV detector coupled to tandem mass spectrometry (UPLC-MS/MS) using a Waters TQD system. Spectra were acquired simultaneously in positive and negative ionisation modes from 50 to 600 m/z and in UV mode (scan 254 nm). MS analysis was performed with an ESI source. Separation was achieved using a Zorbax C18 (5 µm, 4.6 mm × 150 mm) column following a 0.6 mL/min flow rate. The isocratic flow consisted of 30% from mobile phase A (0.1% formic acid in water) and 70% from mobile phase B (0.1% formic acid in acetonitrile), respectively. All the evaluated compounds were ≥ 92% pure. Important to mention that no unexpected

or unusually high safety hazards were encountered during the synthetic procedures..

Experimental Section and synthesis:

Reaction Scheme: 1

Baeyer - Villiger Rearrangement (i) & Hydrolysis (ii) (a):

To a stirred solution of 3,4,5-trimethoxybenzaldehyde (50.96 mmol) and sodium bicarbonate (254.48 mmol) in DCM at 0 °C, was added m-CPBA (101.93 mmol) portion wise and the reaction was stirred for 16 h at room temperature. A saturated solution of $Na_2S_2O_3$ was added to the reaction and extracted three times with DCM. The combined organic layers were washed with water, and the saturated solution of sodium bicarbonate, dried over anhydrous sodium sulphate and concentrated to get crude, which was further taken in methanol and K_2CO_3 (152.9 mmol) was added. The reaction mixture was stirred for 16 hours at room temperature. Solvent was removed under vacuum; crude was taken in a saturated solution of ammonium chloride and extracted with ethyl acetate. The organic layer was dried over anhydrous sodium sulphate and concentrated to get crude, which was purified by column chromatography to give the title compound (yield: 53.3%).

Friedel craft Acylation (b):

To a stirred solution of 3,4,5-trimethoxyphenol (27.14 mmol) in acetic acid at room temperature was added $BF_3.Et_2O$ (15 mL) and the reaction was stirred for 3 hours at 90 °C. The reaction mixture was cooled to room temperature and diluted with ice-cold water, followed by extraction using ethyl acetate. The combined organic layers were dried over anhydrous sodium sulphate and concentrated to get crude, which was purified by column chromatography to get the title compound as a light-yellow liquid product (yield: 74.9%).

Claisen-Schmidt condensation (c):

To a stirred solution of acetophenone (4.42 mmol) in ethanol, were added potassium hydroxide (22.1 mmol) and benzaldehyde (6.63 mmol) at room temperature. The reaction was stirred for 16 hours at room temperature. Solvent was removed under vacuum, and residue was taken in water. The aqueous mixture was acidified using diluted hydrochloric acid and extracted twice with ethyl acetate. The combined organic layers were dried over anhydrous

sodium sulphate and concentrated to get crude, which was purified by column chromatography to get the title compound as a light-yellow solid product (yield: 81.15%).

Claisen-Schmidt condensation (d):

To a stirred solution of acetophenone (12.03 mmol) at -78 °C, was added 2M LDA (30 mmol) and the reaction was stirred for 15 minutes at the same temperature. Dodecanal (18.05 mmol) was added dropwise to the above reaction mixture and stirred it for 2h at -78 °C followed by stirring at room temperature for 16 h before it was quenched by a saturated solution of ammonium chloride and extracted with ethyl acetate. The combined organic layers were dried over anhydrous sodium sulphate and concentrated to get crude, which was purified by column chromatography to get the title compound as an off-white solid product.

Cyclization (e):

To a stirred solution of intermediate-1(2.56 mmol) in DMSO was added Iodine (0.384 mmol) and stirred for 8h at 100 °C. The reaction was cooled to room temperature and poured onto ice cold water and extracted with Ethyl acetate. The combined organic layers were dried over anhydrous Sodium sulphate and concentrated. The obtained crude was purified by column chromatography to get the title compound as yellow solid product.

Demethylation (f):

To a stirred solution of Intermediate-6 (8.23 mmol) in acetic acid was added hydrobromic acid (47%, 9 mL). The reaction was stirred for 18 hours at 90°C. The reaction mixture was concentrated in a vacuum. The obtained crude was purified by column chromatography to get the title compound as a light brown solid product.

2-([1,1'-biphenyl]-4-yl)-5,6,7-trihydroxy-4H-chromen-4-one (FNDR-10131)

Compound 1 **(FNDR-10131)** was synthesized from 1-(6-hydroxy-2,3,4-trimethoxyphenyl) ethan-1-one and 4-phenylbenzaldehyde by following the similar procedure described in synthetic scheme 1. ^{1}H NMR(DMSO,300MHz) δ 12.68(s, 1H), 10.58(s, 1H), 8.82(s, 1H), 8.15-8.17(d, 2H), 7.86-7.88(d, 2H), 7.77-7.79(d, 2H), 7.50-7.53(t, 2H), 7.41-7.45(t, 1H), 7.00(s, 1H), 6.65(s, 1H) LC/MS (ESI-MS) m/z 347 (M+1).

2-(4-hexylphenyl)-5,6,7-trihydroxy-4H-chromen-4-one (FNDR-10132)

Compound 2 **(FNDR-10132)** was synthesized from 1-(6-hydroxy-2,3,4-trimethoxyphenyl) ethan-1-one and 4-hexylbenzaldehyde by following the similar procedure described in synthetic scheme 1. ^{1}H NMR(DMSO,300MHz) δ12.70(s, 1H), 10.54(s, 1H), 8.79(s, 1H), 7.96-7.98(d, 2H), 7.38-7.40(d, 2H), 6.88(s, 1H), 6.61(s, 1H), 2.67-2.68(t, 2H), 1.58-1.61(m, 2H), 1.28(m, 6H), 0.84-0.87(t, 3H) LC/MS (ESI-MS) m/z 355 (M+1).

5,6,7-trihydroxy-2-(4-morpholinophenyl)-4H-chromen-4-one(FNDR-10133)

Compound 3 **(FNDR-10133)** was synthesized from 1-(6-hydroxy-2,3,4-trimethoxyphenyl) ethan-1-one and 4-morpholinobenzaldehyde by following the similar procedure described in synthetic scheme 1. ^{1}H NMR(DMSO,300MHz) δ12.86(s, 1H), 10.42(s, 1H), 8.72(s, 1H), 7.90-7.92(d, 2H), 7.04-7.07(d, 2H), 6.75(s, 1H), 6.57(s, 1H), 3.73-3.75(t, 2H), 3.28-3.30(t, 2H) LC/MS (ESI-MS) m/z 356 (M+1).

5,6,7-trihydroxy-2-(4-(piperidin-1-yl)phenyl)-4H-chromen-4-one(FNDR-10136)

Compound 4 **(FNDR-10136)** was synthesized from 1-(6-hydroxy-2,3,4-trimethoxyphenyl) ethan-1-one and 4-(piperidin-1-yl)benzaldehyde by following the similar procedure described in synthetic scheme 1. ^{1}H NMR(DMSO,300MHz) δ12.91(s, 1H), 10.38(bd, 1H), 8.52-9.05(m, 1H), 7.86-7.88(d,2H), 7.01-7.03(d, 2H), 6.70(s, 1H), 6.56(s, 1H), 3.37(bs, 5H), 1.56-1.64(m, 6H) LC/MS (ESI-MS) m/z 354 (M+1).

5,6,7-trihydroxy-2-undecyl-4H-chromen-4-one (FNDR-10142)

Compound 5 **(FNDR-10142)** was synthesized from 1-(6-hydroxy-2,3,4-trimethoxyphenyl) ethan-1-one and dodecanal by following the similar procedure described in synthetic scheme 1. ^{1}H NMR(DMSO,300MHz) δ 12.65(s, 1H), 10.41(bs, 1H), 8.69(bs, 1H), 6.41(s, 1H), 6.11(s, 1H), 2.57-2.61(t, 2H), 1.61-1.64(m, 2H), 1.23-1.28(bs, 20H), 0.82-0.86(t, 3H) LC/MS (ESI-MS) m/z 349 (M+1).

Scheme II

Claisen-Schmidt condensation (a) & Cyclization (b):

The preparation procedure for Intermediate-5 and cyclization follows the same reaction as scheme I.

4-oxo-2-phenyl-4H-chromene-6-carboxylic acid (FNDR-10130)

Compound 6 **(FNDR-10130)** was synthesized from 3-acetyl-4-hydroxybenzoic acid and benzaldehyde by following the similar procedure described in synthetic scheme 2. ^{1}HNMR (DMSO,300MHz) δ8.58-8.59(d,1H),8.30-8.37(dd,1H), 8.14-8.16(dd,2H), 7.90-7.92 (d,1H), 7,58-7.66(m,3H) LC/MS (ESI-MS) m/z 267 (M+1).

2- ([1,1'-biphenyl]-4-yl)-4-oxo-4H-chromene-6-carboxylic acid (FNDR-10137)

Compound 7 **(FNDR-10137)** was synthesized from 3-acetyl-4-hydroxybenzoic acid and [1,1'-biphenyl]-4-carbaldehyde by following the similar procedure described in synthetic scheme 1. ^{1}H NMR(DMSO,300MHz) δ 13.37(bs, 1H), 8.60(s, 1H), 8.31-8.34(d, 1H), 8.22-8.25(d, 2H), 7.90-7.94(t, 3H), 7.79-7.80(d, 2H), 7.50-7.54(t, 2H), 7.42-7.46(t, 1H), 7.19(s, 1H), LC/MS (ESI-MS) m/z 343.4 (M+1).

2-(4-morpholinophenyl)-4-oxo-4H-chromene-6-carboxylic acid (FNDR-10138)

Compound 8 **(FNDR-10138)** was synthesized from 3-acetyl-4-hydroxybenzoic acid and 4-morpholinobenzaldehyde by following the similar procedure described in synthetic scheme 2.^{1}H NMR (DMSO,300MHz) δ 13.29(bs, 1H), 8.56-8.57(d, 1H), 8.26-8.29(d, 1H), 7.99-8.01(d, 2H), 7.84-7.87(d, 1H), 7.08-7.11(d, 2H), 6.95(s, 1H), 3.74-3.77(t, 4H), 3.30(bs, 4H) LC/MS (ESI-MS) m/z 352 (M+1).

4-oxo-2-(4-(piperidin-1-yl)phenyl)-4H-chromene-6-carboxylic acid (FNDR-10139)

Compound 9 **(FNDR-10139)** was synthesized from 3-acetyl-4-hydroxybenzoic acid and 4-(piperidin-1-yl) benzaldehyde by following the similar procedure described in synthetic scheme 2. ^{1}H NMR(DMSO,300MHz) δ 8.55-8.56(s, 1H), 8.25-8.28(dd, 1H), 7.93.7.95(d, 2H), 7.83-7.85(d, 1H), 7.04-7.06(d, 2H), 6.89(s, 1H), 3.35(bs, 4H), 1.60(bs, 6H) LC/MS (ESI-MS) m/z 350 (M+1).

2-(4-cyclohexylphenyl)-4-oxo-4H-chromene-6-carboxylic acid (FNDR-10999)

Compound 10 **(FNDR-10999)** was synthesized from 3-acetyl-4-hydroxybenzoic acid and 4-cyclohexylbenzaldehyde by following the similar procedure described in synthetic scheme 2.^{1}H NMR(DMSO,300MHz) δ 8.69-8.70(d, 1H), 8.18-8.21(dd, 1H), 7.70-7.72(d, 2H), 7.49-7.51(d, 1H), 7.19-7.22(d, 2H), 6.67(s, 1H), 2.46(bs, 1H), 1.67-1.73(m, 4H), 1.22-1.29(m, 4H), 1.06-1.12(m, 2H) LC/MS (ESI-MS) m/z 349 (M+1).

2-(4-(diethylamino) phenyl)-4-oxo-4H-chromene-6-carboxylic acid (FNDR-11001).

Compound 11 **(FNDR-11001)** was synthesized from 3-acetyl-4-hydroxybenzoic acid and 4-(diethylamino) benzaldehyde by following the similar procedure described in synthetic scheme 2. ^{1}H NMR(DMSO,300MHz) δ 13.29(bs, 1H), 8.55-8.56(d, 1H), 8.25-8.27(dd, 1H), 7.92-7.94(d, 2H), 7.81-7.83(d, 1H), 6.85(s, 1H), 6.79-6.81(d, 2H), 3.42-3.48(q, 4H), 1.12-1.16(t, 6H) LC/MS (ESI-MS) m/z 349 (M+1).

2-(4-hexylphenyl)-4-oxo-4H-chromene-6-carboxylic acid (FNDR-11009)

Compound 12 **(FNDR-11009)** was synthesized from 3-acetyl-4-hydroxybenzoic acid and 4-hexyl benzaldehyde by following the similar procedure described in synthetic scheme 2.^{1}H NMR(DMSO,300MHz) δ 8.58-8.59(d,1H), 8.30-8.32(dd,1H), 8.04-8.06(d,2H), 7.87-7.89(d,1H), 7.41-7.43(d,2H),7.08(s,1H),2.65-2.69(t,2H), 1.57-1.62(m,2H), 1.22-1.28(m,8H),0.84-0.87(t,3H). LC/MS (ESI-MS) m/z 351 (M+1).

4-oxo-2-(4-(piperidin-1-yl)phenyl)-4H-chromene-7-carboxylic acid (FNDR-11003)

Compound 13 **(FNDR-11003)** was synthesized from 4-acetyl-3-hydroxybenzoic acid and 4-(piperidin-1-yl) benzaldehyde by following the similar procedure described in synthetic scheme 2.^{1}H NMR(DMSO,300MHz) δ 8.55-8.56(d,1H), 8.25-8.28(dd,1H), 7.93-7.95(d,2H),7.82-7.84(d,1H),7.03-7.06(d,2H),6.89(s,4H),1.60(s,6H), LC/MS (ESI-MS) m/z 351 (M+1).

2-(4-cyclohexylphenyl)-4-oxo-4H-chromene-7-carboxylic acid (FNDR-11012).

Compound 14 **(FNDR-11012)** was synthesized from 4-acetyl-3-hydroxybenzoic acid and 4-cyclohexyl benzaldehyde by following the similar procedure described in synthetic scheme 2.^{1}H NMR(DMSO,300MHz) δ 8.69-8.70 (d,1H),8.18-8.21(dd,1H),7.70-7.72(d,2H),7.49-7.51(d,1H),7.19-7.22(d,2H),6.67(s,1H),2.46(s,1H),1.67-1.73(m,4H),1.22-1.29(m,4H),1.06-1.12(,2H), LC/MS (ESI-MS) m/z 349 (M+1).

2-(4-(diethylamino)phenyl)-4-oxo-4H-chromene-7-carboxylic acid (FNDR-11014).

Compound 15 **(FNDR-11014)** was synthesized from 4-acetyl-3-hydroxybenzoic acid and 4-(diethylamino)benzaldehyde by following the similar procedure described in synthetic scheme 2.^{1}H NMR(DMSO,300MHz) δ 8.55-8.56(d,1H),8.25-8.27(d,1H),7.92-7.94(d,2H),7.83-7.81(d,1H),6.79-6.81(d,2H), 6.85(s,1H), 3.42-3.48(m,4H), LC/MS (ESI-MS) m/z 338 (M+1).

Reaction Scheme:3

Formation of sulfonic ester (a):

To the stirred solution of 3-(methylthio)propan-1-ol (200 mmol) and Tosyl chlride (200 mmol) in Dichloromethane (5 V) at -20 °C was added dropwise pyridine (500 mmol). The reaction mixture was allowed to react at -20 °C overnight and then at room temperature for 2 h. The reaction mixture was quenched with water and extracted with dichloromethane. The combined organic layers were dried over anhydrous sodium sulphate and concentrated. The obtained crude was dissolved in dichloromethane followed by precipitation with hexane The resulting product was decanted, and solvents was removed under vacuum pressure to obtain the desired product. Colorless oil, yield 96%:

Oxidation of Thioethers to Sulfoxides and Sulfones (b):

To the stirred solution 3-(Methylthio) propyl 4-methylbenzenesunfonate (19 mmol) was dissolved in methanol (5 V) and the mixture was cooled to 0 to 5 °C. While cooling to 0 to 5°C oxone (76 mmol) dissolved in water (5 V) was added dropwise over 1 hr. After stirring at 0 to 5 °C for 1 h. the mixture was stirred at room temperature for 12 hr. The reaction mixture was concentrated under vacuum pressure. The obtained crude was dissolved in water and extracted with ethyl acetate. The combined organic layer was dried over anhydrous sodium sulphate and concentrated under vacuum pressure to obtain the desired product 3-(methylsulfonyl) propyl 4-methylbenzenesunfonate (Yield 87%)

Alkylation (c):

To the stirred solution of 3-(methylsulfonyl) propyl 4-methylbenzenesunfonate (13 mmol) in DMF (5 V) followed by K_2CO_3(11 mmol) & 4-hydroxybenzaldehyde (13 mmol) was added into the reaction mixture and stirred overnight at RT. The reaction mixture was poured into ice-cold water and extracted with ethyl acetate. The combined organic layer was dried over sodium sulphate and concentrated.

Claisen-Schmidt condensation (d) &Cyclization (e):

The preparation procedure for Intermediate-1 and Cyclization follows the same reaction as scheme I.

2-(4-butoxyphenyl)-4-oxo-4H-chromene-6-carboxylic acid (FNDR-11000)

Compound 16 **(FNDR-11000)** was synthesized from 3-acetyl-4-hydroxybenzoic acid and 4-butoxybenzaldehyde by following the similar procedure described in synthetic scheme 3. ^{1}H NMR(DMSO,300MHz) δ 13.28(bs, 1H), 8.57-8.58(d, 1H), 8.28-8.31(dd, 1H), 8.07-8.09(d, 2H), 7.86-7.88(d, 1H), 7.11-7.13(d, 2H), 7.02(s, 1H), 4.07-4.10(t, 2H), 1.70-1.77(m, 2H), 1.41-1.50(m, 2H), 0.93-0.96(t, 3H) LC/MS (ESI-MS) m/z 339 (M+1).

2-(4-(diethylamino)phenyl)-4-oxo-4H-chromene-6-carboxylic acid (FNDR-11011)

Compound 17 **(FNDR-11011)** was synthesized from 3-acetyl-4-hydroxybenzoic acid and 4-(diethylamino) benzaldehyde by following the similar procedure described in synthetic scheme 3.^{1}H NMR(DMSO,300MHz) δ 13.29(bs, 1H), 8.55-8.56(d, 1H), 8.25-8.27(dd, 1H), 7.92-7.94(d, 2H), 7.81-7.83(d, 1H), 6.85(s, 1H), 6.79-6.81(d, 2H), 3.42-3.48(q, 4H), 1.12-1.16(t, 6H) LC/MS (ESI-MS) m/z 338 (M+1).

2-(4-(3-(methylsulfonyl) propoxy)phenyl)-4-oxo-4H-chromene-6-carboxylic acid (FNDR-11096)

Compound 18 **(FNDR-11096)** was synthesized from 3-acetyl-4-hydroxybenzoic acid and 4-(3-(methylsulfonyl) propoxy)benzaldehyde by following the similar procedure described in synthetic scheme 3. ^{1}H NMR (DMSO,300MHz)

δ 13.36(bs, 1H), 8.58(s, 1H), 8.28-8.31(d, 1H), 8.10-8.13(d, 2H), 7.87-7.90(d, 2H), 7.14-7.17(d, 2H), 7.05(s, 1H), 4.20-4.24(t, 2H), 3.28-3.31(t, 2H), 3.04(s, 3H), 2.16-2.21(m, 2H) LC/MS (ESI-MS) m/z 403 (M+1).

2-(4-butoxyphenyl)-4-oxo-4H-chromene-7-carboxylic acid (FNDR-11013)

Compound 19 **(FNDR-11013)** was synthesized from 4-acetyl-3-hydroxybenzoic acid and 4-butoxybenzaldehyde by following the similar procedure described in synthetic scheme 3. ^{1}HNMR(DMSO,300MHz) δ 8.57-8.58 (d,1H),8.28-8.31(dd,1H),8.07-8.09(d,2H),7.86-7.88(d,1H),7.11-7.13(d,2H),7.02(s,1H)4.07-4.10(t,2H),1.70-1.77(m,2H),1.41-1.50(m2H),0.93-0.96(t,3H),LC/MS (ESI-MS) m/z 339 (M+1).

Scheme IV:

Baeyer - Villiger Rearrangement (i) & Hydrolysis (ii) (a) & Friedel craft Acylation (b):

The preparation procedure for intermediate-7 and intermediate-8 follows the same reaction as scheme I.

Acylation of Ketones and Nitriles by Carboxylic Esters (c):

Sodium metal (9.14 g, 397.82 mmol) was added lot wise to a stirred ethanol (300 mL) under N_2 atmosphere. Once Sodium metal was completely dissolved then 1-(6-hydroxy-2,3,4-trimethoxyphenyl)ethan-1-one (9 g, 39.78 mmol) and diethyl oxalate (34.88 g, 238.69 mmol) were added to the above mixture. The reaction was refluxed for 16h before it was cooled to room temperature and quenched with crushed ice. The aqueous mixture was acidified using dilute Hydrochloric acid and extracted 3 times with Ethyl acetate. The combined organic layers were dried over anhydrous Sodium sulphate and concentrated to get crude (15 g) which was used next stage without further purification.

Formation of Oxygen Heterocycles (d)

To a stirred solution of ethyl 4-(6-hydroxy-2,3,4-trimethoxyphenyl)-2,4-dioxobutanoate (10 g, 30.64 mmol) in Ethanol (15 mL) at room temperature, was added conc. HCl (30 mL, 3 Volumes) and the reaction was refluxed for 16h. The Reaction mixture was poured on crushed ice followed by extraction using Ethyl acetate. The combined organic layers were dried over anhydrous Sodium sulphate and concentrated to get crude which was purified by column chromatography to get title compound as brown semi-solid product (3.5 g, yield: 37.7%).

Reduction (e):

To a stirred solution of ethyl 5,6,7-trimethoxy-4-oxo-4H-chromene-2-carboxylate (5.5 g, 17.84mmol) in THF:Ethanol (1:1, 200 mL) at 0°C, was added Sodium borohydride (1.34 g, 35.68 mmol) portion wise and the reaction was stirred for 3h at 0-5°C. Reaction mixture was poured on crushed ice then acidified with dil. HCl and extracted 3 times using Ethyl acetate. The combined organic layers were dried over anhydrous Sodium sulphate and concentrated to get crude which was washed with Diethyl ether to get title compound as off-white solid product (2.5 g, yield: 52.68%).

Formation of Mesylates (f):

To a stirred solution of 2-(hydroxymethyl)-5,6,7-trimethoxy-4H-chromen-4-one (2.5 g, 9.38 mmol) in DCM (30 mL) at 0°C, was added Triethylamine (6.54 mL, 46.94 mmol) followed by dropwise addition of Methanesulfonyl chloride (2.18 mL, 28.16 mL) and the reaction was stirred for 3h at 0-5°C. The reaction mixture was quenched with water and extracted 3 times with Dichloromethane. The combined organic layers were dried over anhydrous Sodium sulphate and concentrated to get thick yellow oil as crude (3.0 g) product which was used next stage without further purification.

Formation of Azide (g):

To a stirred solution of (5,6,7-trimethoxy-4-oxo-4H-chromen-2-yl)methyl methanesulfonate (1.6 g, 4.64 mmol) in DMF (10 mL) at 0°C, was added Sodium azide (0.9 g, 13.94 mmol) and the reaction was stirred for 2h at room temperature. The Reaction mixture was poured on crushed ice followed by extraction using Ethyl acetate. The combined organic layers were dried over anhydrous Sodium sulphate and concentrated to get crude which was purified by column chromatography to get title compound as brown oil product (1.1 g, yield: 81.48%).

Staudinger Reduction (h):

To a stirred solution of 2-(azidomethyl)-5,6,7-trimethoxy-4H-chromen-4-one (1.1 g, 3.77 mmol) in THF: Diethyl ether (1:1, 50 mL) at 0°C, was added PPH_3 (2.97 g, 11.33 mmol) and the reaction was stirred for 10 min at 0°C and 1h at room temperature. 6N HCl was added to the reaction mixture and stirred for 16h at room temperature. The reaction was diluted with water and separated aqueous layer was washed with Ethyl acetate. The combined organic layers were dried over anhydrous Sodium sulphate and concentrated to get crude which was triturated with Acetonitrile and washed with Diethyl acetate to get yellowish solid product as crude (0.5 g, 43.85%).

Demethylation (i):

To a stirred solution of 2-(aminomethyl)-5,6,7-trihydroxy-4H-chromen-4-one hydrobromide (0.19 g, 4.64 mmol) in Acetic acid (50 mL) at, was added 47% Hydrobromic acid (30 mL) and the reaction was reflux for 48h. The reaction mixture was concentrated under vacuum and residue was washed with Diethyl ether and dried to get brown color solid as product (0.95 g, 67.37%).

Amide Formation(j):

To a stirred solution of 2-(aminomethyl)-5,6,7-trihydroxy-4H-chromen-4-one hydrobromide (0.19 g, 0.62 mmol) in DMF (5 mL), were added HATU (0.261, 0.87 mmol), DIPEA (0.323 g, 2.5 mmol) and acid (0.082g, 0.49 mmol). The reaction mixture was stirred for 16h at room temperature. The reaction was diluted with water and separated aqueous layer was washed with Ethyl acetate. The combined organic layers were dried over anhydrous Sodium sulphate and concentrated to get crude which purified by Prep HPLC to get title compound as off-white solid product (0.015g, 6.52%).

4-phenyl-N-((5,6,7-trihydroxy-4-oxo-4H-chromen-2-yl)methyl)butanamide (FNDR-10143)

Compound 20 **(FNDR-10143)** was synthesized from 2-(aminomethyl)-5,6,7-trihydroxy-4H-chromen-4-one hydrochloride and 4-phenylbutanoic acid by following the similar procedure described in synthetic scheme 4. ^{1}H NMR(DMSO,300MHz) δ12.53(s, 1H), 10.50(bs, 1H), 8.75(bs, 1H), 8.3(t, 1H), 7.26-7.29(t, 2H), 7.17-7.19(d, 3H), 6.42(s, 1H), 6.06(s, 1H), 4.20-4.22 (d, 2H), 2.57-2.59(t, 2H), 2.18-2.22(t, 2H), 1.79-1.86(m, 2H)LC/MS (ESI-MS) m/z 370 (M+1).

3-(3-methoxyphenyl)-N-((5,6,7-trihydroxy-4-oxo-4H-chromen-2-yl)methyl)propanamide (FNDR-10146)

Compound 21 **(FNDR-10146)** was synthesized from 2-(aminomethyl)-5,6,7-trihydroxy-4H-chromen-4-one hydrochloride and 3-(3-methoxyphenyl) propanoic acid by following the similar procedure described in synthetic scheme 4. ^{1}H NMR(DMSO,300MHz) δ12.55(s, 1H), 10.51(bs, 1H), 8.76(bs, 1H), 8.49-8.51(t, 1H), 7.14-7.18(t, 1H), 6.72-6.78(m, 3H), 6.41(s, 1H), 5.99(s, 1H),4.20-4.21(d, 2H), 3.71(s, 3H), 2.80-2.83(t, 2H)LC/MS (ESI-MS) m/z 386 (M+1).

4-butoxy-N-((5,6,7-trihydroxy-4-oxo-4H-chromen-2-yl)methyl)benzamide(FNDR-10148)

Compound 22 **(FNDR-10148)** was synthesized from 2-(aminomethyl)-5,6,7-trihydroxy-4H-chromen-4-one hydrochloride and 4-butoxybenzoic acid by following the similar procedure described in synthetic scheme 4. ^{1}H NMR(DMSO,300MHz) δ12.53(s, 1H), 10.49(bs, 1H), 8.98(s, 1H), 8.77(bs, 1H), 7.86-7.88(d, 2H), 7.01-7.02(d, 2H), 6.43(s, 1H), 6.08(s, 1H), 4.39(bs, 2H), 4.03(bs, 2H), 1.70(bs, 2H), 1.43-1.44(m, 2H), 0.93(bs, 3H)LC/MS (ESI-MS) m/z 400 (M+1).`

5-phenyl-N-((5,6,7-trihydroxy-4-oxo-4H-chromen-2-yl)methyl)pentanamide(FNDR-10149)

Compound 23 (FNDR-10149) was synthesized from 2-(aminomethyl)-5,6,7-trihydroxy-4H-chromen-4-one hydrochloride and 5-phenylpentanoic acid by following the similar procedure described in synthetic scheme 4. ^{1}H NMR(DMSO,300MHz) δ12.54(s, 1H), 10.51(s, 1H), 8.76(s, 1H), 8.46-8.49(t, 1H), 7.23-7.27(t, 2H), 7.13-7.17(t, 3H), 6.41(s, 1H), 6.05(s, 1H), 4.19-4.21(d, 2H), 2.55-2.59(t, 2H), 2.19-2.23(t, 2H), 1.54-1.55(t, 4H),LC/MS (ESI-MS) m/z 384 (M+1).`

6-bromo-N-((5,6,7-trihydroxy-4-oxo-4H-chromen-2-yl)methyl)-2-naphthamide (FNDR-10150)

Compound 24 **(FNDR-10150)** was synthesized from 2-(aminomethyl)-5,6,7-trihydroxy-4H-chromen-4-one hydrochloride and 6-bromo-2-naphthoic acid by following the similar procedure described in synthetic scheme 4. ^{1}H NMR(DMSO,300MHz) δ 12.53(s, 1H), 10.54(bs, 1H), 9.34-9.37(t, 1H), 8.79(bs, 1H), 8.55(s, 1H), 8.31(s, 1H), 8.02-8.04(d, 3H), 7.72-7.75(d, 2H), 6.45(s, 1H), 6.18(s, 1H), 4.47-4.48(d, 2H)LC/MS (ESI-MS) m/z 457 (M+1).

^{1}H NMR spectra and LC-MS spectra for the active compounds tested against *P. falciparum*

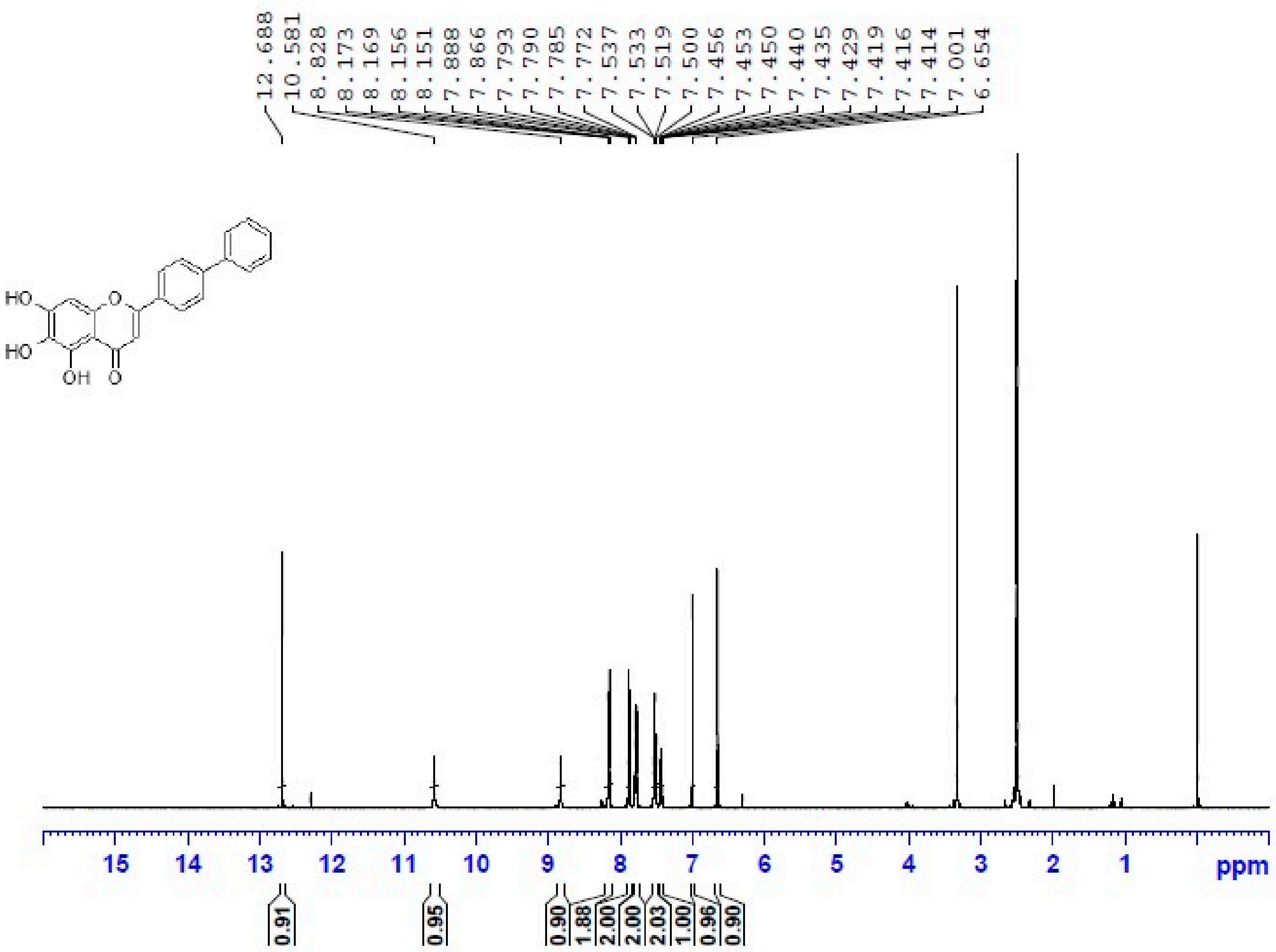

Figure S1 : ^{1}H NMR spectra of compound 1 (FNDR-10131)

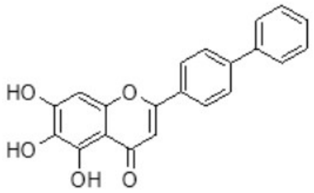

Mol.Wt.:346.33

LC-MS Analysis Report

<Sample Information>

14-05-2018_18.lcd

Sample Name	: Demethylation	Sample ID	: ALS18AF094-0041-30-03
Date Acquired	: 5/14/2018 12:31:00 PM	Date Processed	: 5/14/2018 12:49:00 PM
Method File	: LCMS 01.lcm		

<LC Chromatogram>

14-05-2018_18.lcd

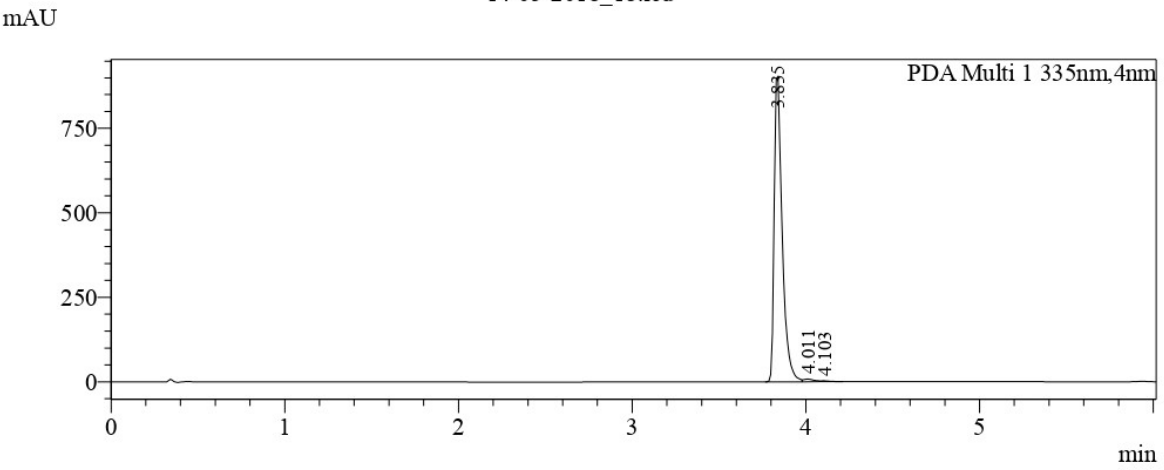

Peak Table 14-05-2018_18.lcd

PDA Ch1 335nm

Peak#	Name	Ret. Time	Area	Area%
1		3.835	2889702	98.592
2		4.011	33677	1.149
3		4.103	7587	0.259
Total			2930965	100.000

Line#:3 R.Time:4.200(Scan#:1261)
MassPeaks:7
Spectrum Mode:Single 4.200(1261) Base Peak:347(573170)
BG Mode:None Segment 1 - Event 1

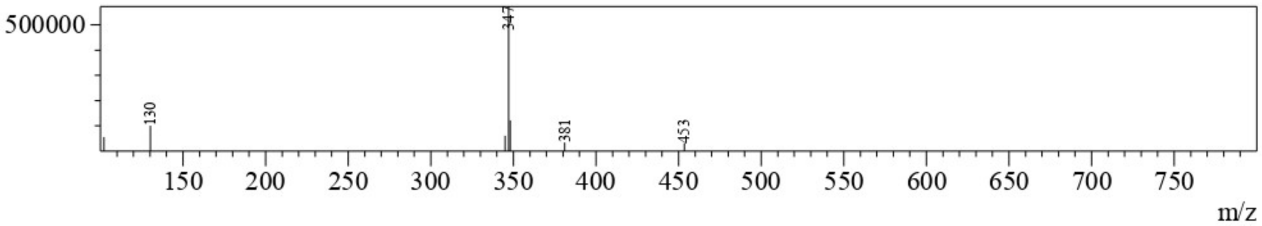

Figure S2 : LC-MS Spectra of compound 1 (FNDR-10131)

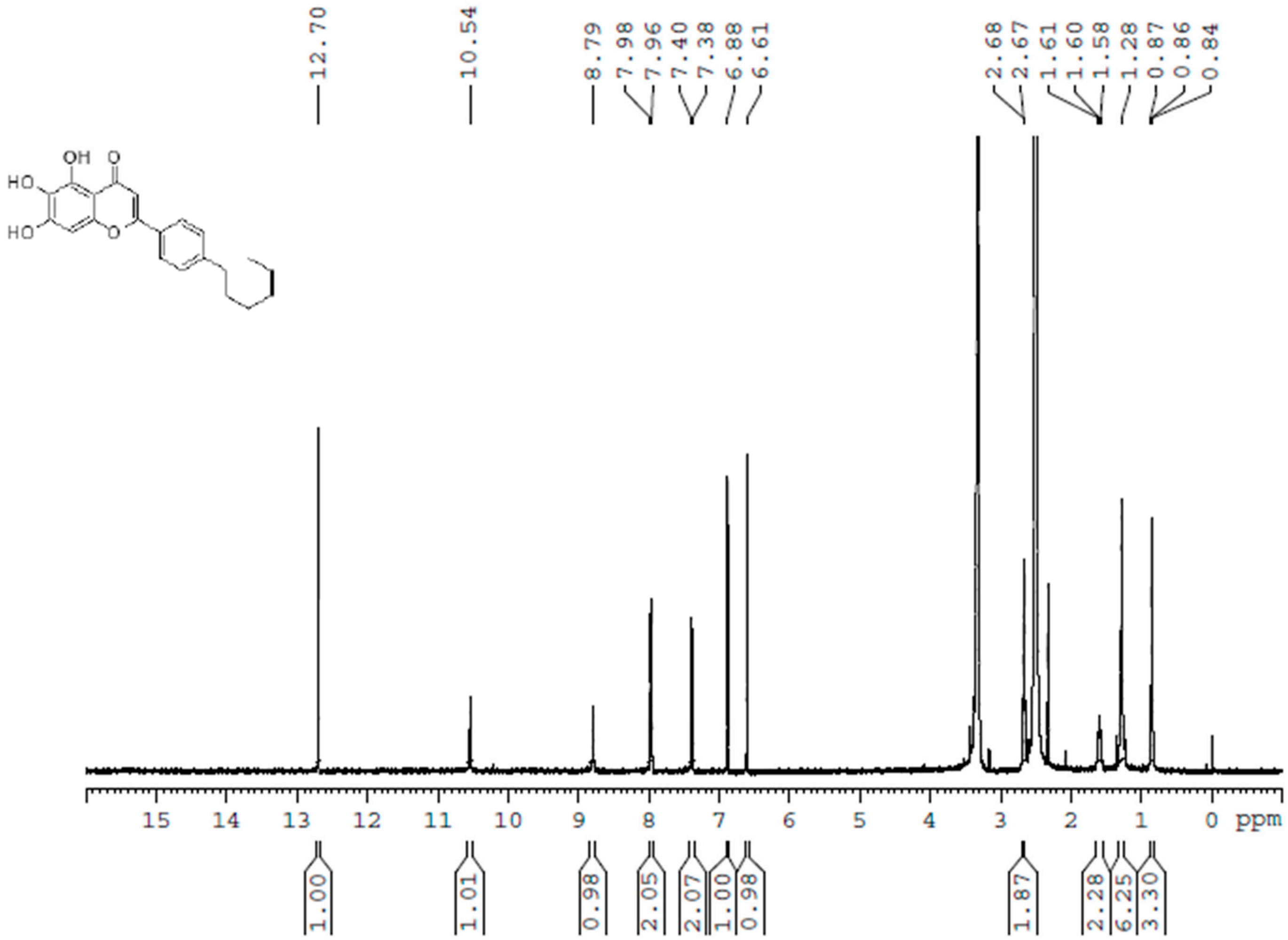

Figure S3: ^{1}H NMR spectra of compound 2 (FNDR-10132)

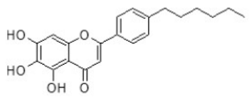

Mol.Wt.:354.39

LC-MS Analysis Report

<Sample Information>

22-05-2018_66.lcd

Sample Name	: Demethylation	Sample ID	: ALS18AF094-0041-43-03
Date Acquired	: 5/22/2018 7:14:50 PM	Date Processed	: 5/23/2018 4:13:57 PM
Method File	: LCMS 01.lcm		

<LC Chromatogram>

22-05-2018_66.lcd

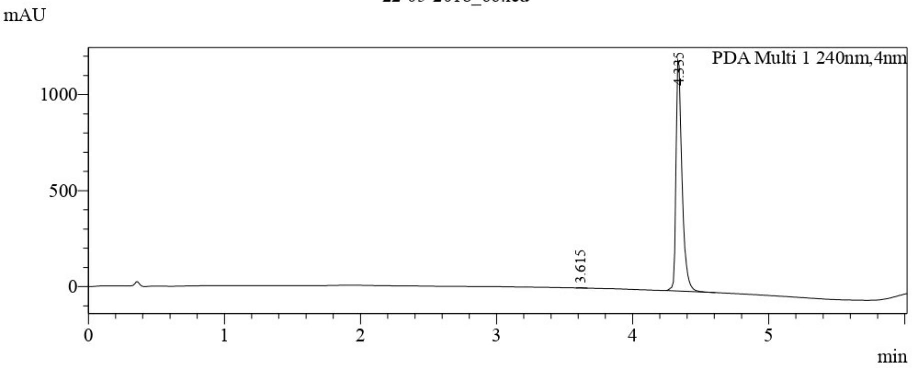

Peak Table 22-05-2018_66.lcd

PDA Ch1 240nm

Peak#	Name	Ret. Time	Area	Area%
1		3.615	2764	0.075
2		4.335	3706444	99.925
Total			3709208	100.000

<MS Spectrum>

22-05-2018_66.lcd

Line#:1 R.Time:4.400(Scan#:1321)
MassPeaks:5
Spectrum Mode:Single 4.400(1321) Base Peak:355(6166462)
BG Mode:None Segment 1 - Event 1

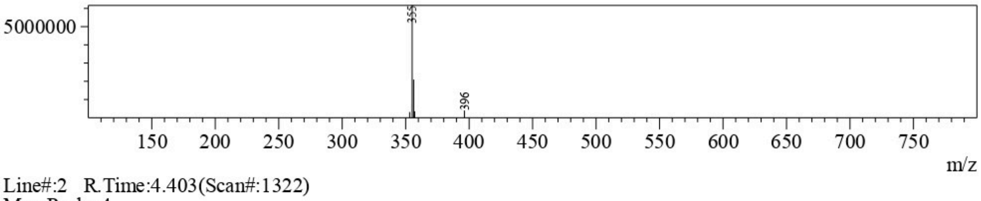

Line#:2 R.Time:4.403(Scan#:1322)
MassPeaks:4
Spectrum Mode:Single 4.403(1322) Base Peak:353(1223995)
BG Mode:None Segment 1 - Event 2

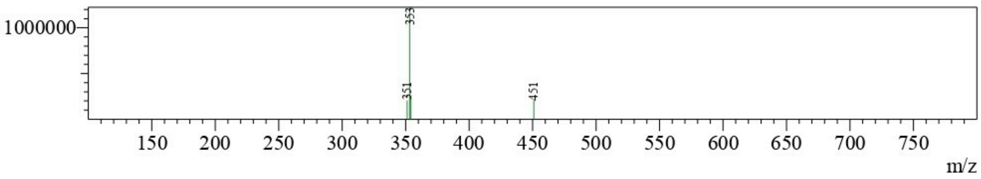

Figure S4: LC-MS spectra of compound 2 (FNDR-10132)

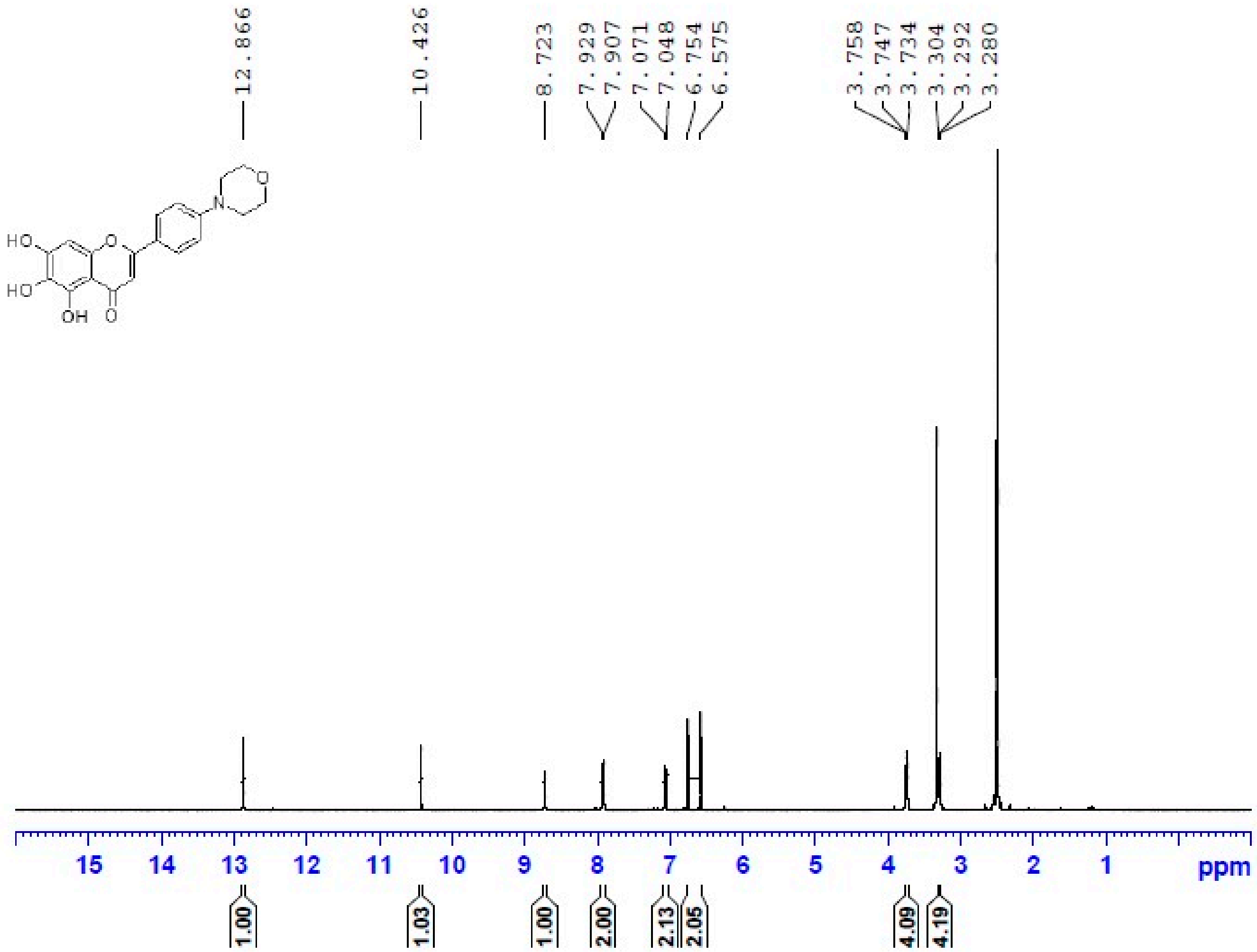

Figure S5: ^{1}H NMR spectra of compound 3 (FNDR-10133)

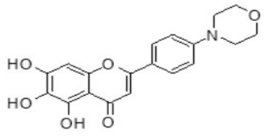

Mol.Wt.:355.34

LC-MS Analysis Report

<Sample Information>

22-05-2018_36.lcd

Sample Name	: Demethylation	Sample ID	: ALS18AF094-0041-39-04
Date Acquired	: 5/22/2018 1:31:27 PM	Date Processed	: 5/22/2018 2:08:26 PM
Method File	: LCMS_01.lcm		

<LC Chromatogram>

22-05-2018_36.lcd

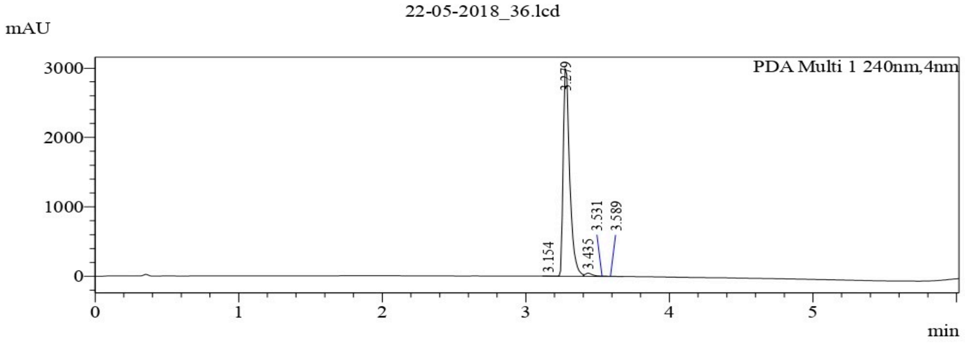

Peak Table 22-05-2018_36.lcd

PDA Ch1 240nm

Peak#	Name	Ret. Time	Area	Area%
1		3.154	7238	0.075
2		3.279	9461480	97.996
3		3.435	164931	1.708
4		3.531	15064	0.156
5		3.589	6278	0.065
Total			9654992	100.000

<MS Spectrum>

22-05-2018_36.lcd

Line#:1 R.Time:3.340(Scan#:1003)
MassPeaks:3
Spectrum Mode:Single 3.340(1003) Base Peak:356(2039699)
BG Mode:None Segment 1 - Event 1

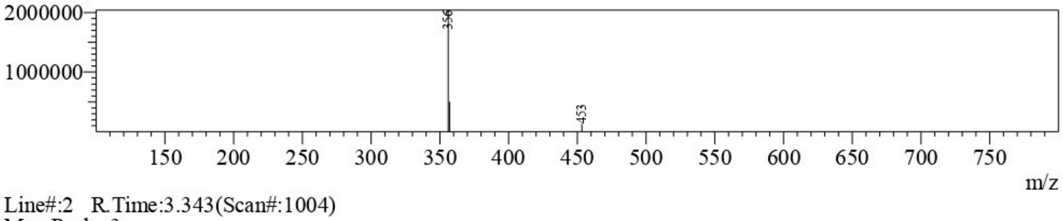

Line#:2 R.Time:3.343(Scan#:1004)
MassPeaks:3
Spectrum Mode:Single 3.343(1004) Base Peak:354(561800)
BG Mode:None Segment 1 - Event 2

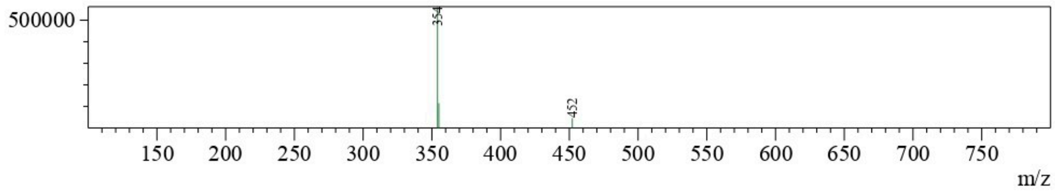

Figure S6: LC-MS spectra of compound 3 (FNDR-10133)

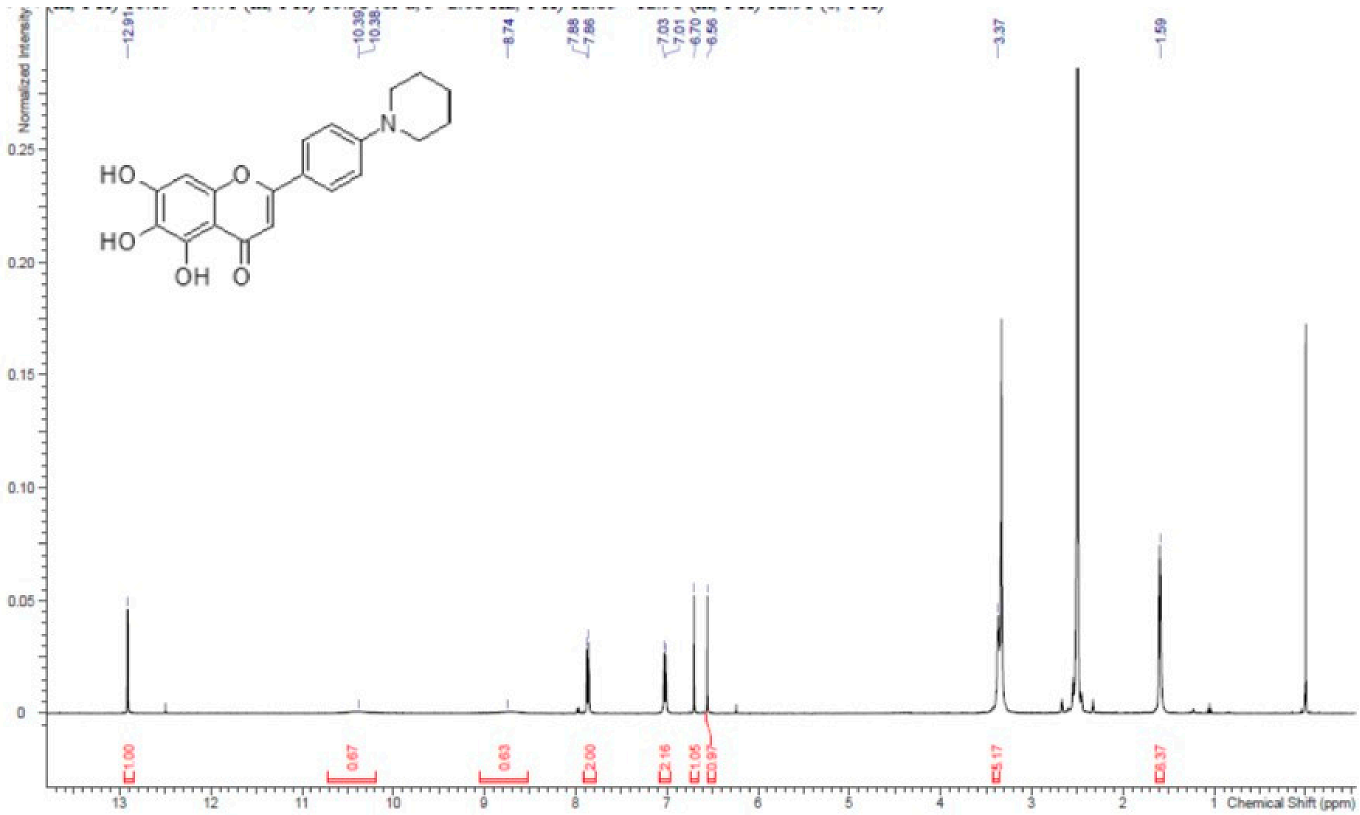

Figure S7: ^{1}H NMR spectra of compound 4 (FNDR-10136)

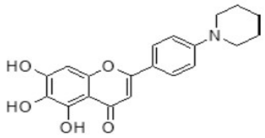

Mol.Wt.:353.36

LC-MS Analysis Report

<Sample Information>

28-05-2018_33.lcd

Sample Name	: Demethylation	Sample ID	: ALS18AF094-0041-38-06
Date Acquired	: 5/28/2018 4:33:54 PM	Date Processed	: 5/29/2018 11:42:45 AM
Method File	: LCMS 01.lcm		

<LC Chromatogram>

28-05-2018_33.lcd

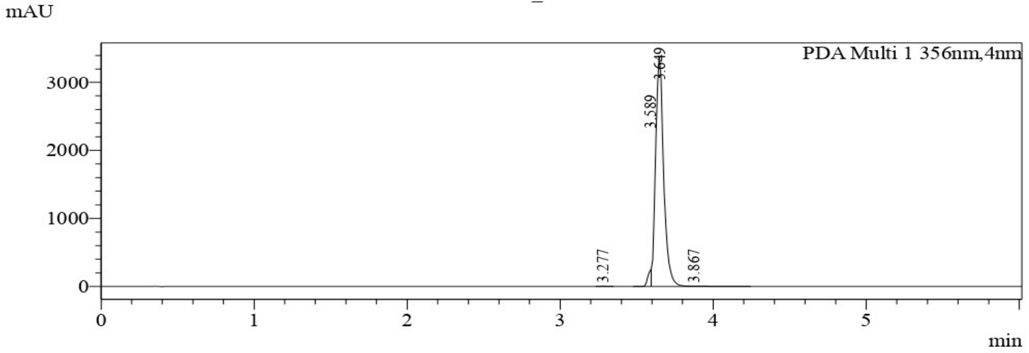

Peak Table 28-05-2018_33.lcd

PDA Ch1 356nm

Peak#	Name	Ret. Time	Area	Area%
1		3.277	11866	0.093
2		3.589	445144	3.489
3		3.649	12270311	96.184
4		3.867	29833	0.234
Total			12757155	100.000

<MS Spectrum>

28-05-2018_33.lcd

Line#:1 R.Time:3.707(Scan#:1113)
MassPeaks:5
Spectrum Mode:Single 3.707(1113) Base Peak:354(6007721)
BG Mode:None Segment 1 - Event 1

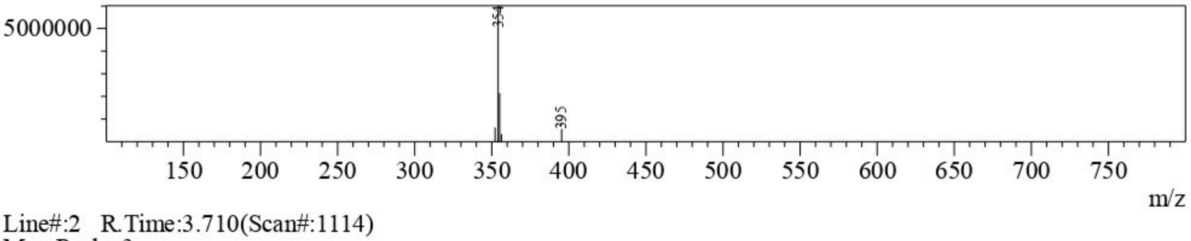

Line#:2 R.Time:3.710(Scan#:1114)
MassPeaks:3
Spectrum Mode:Single 3.710(1114) Base Peak:352(1423380)
BG Mode:None Segment 1 - Event 2

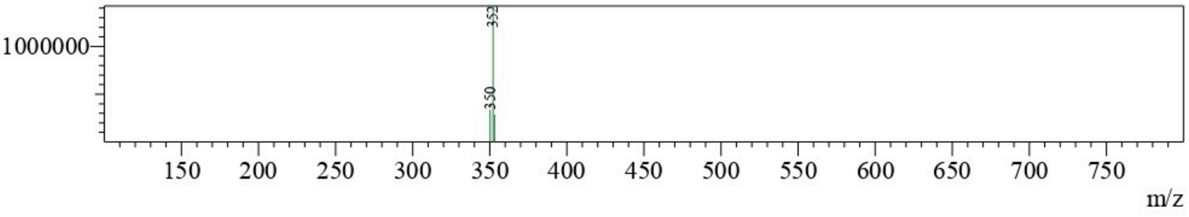

Figure S8: LC-MS spectra of compound 4 (FNDR-10136)

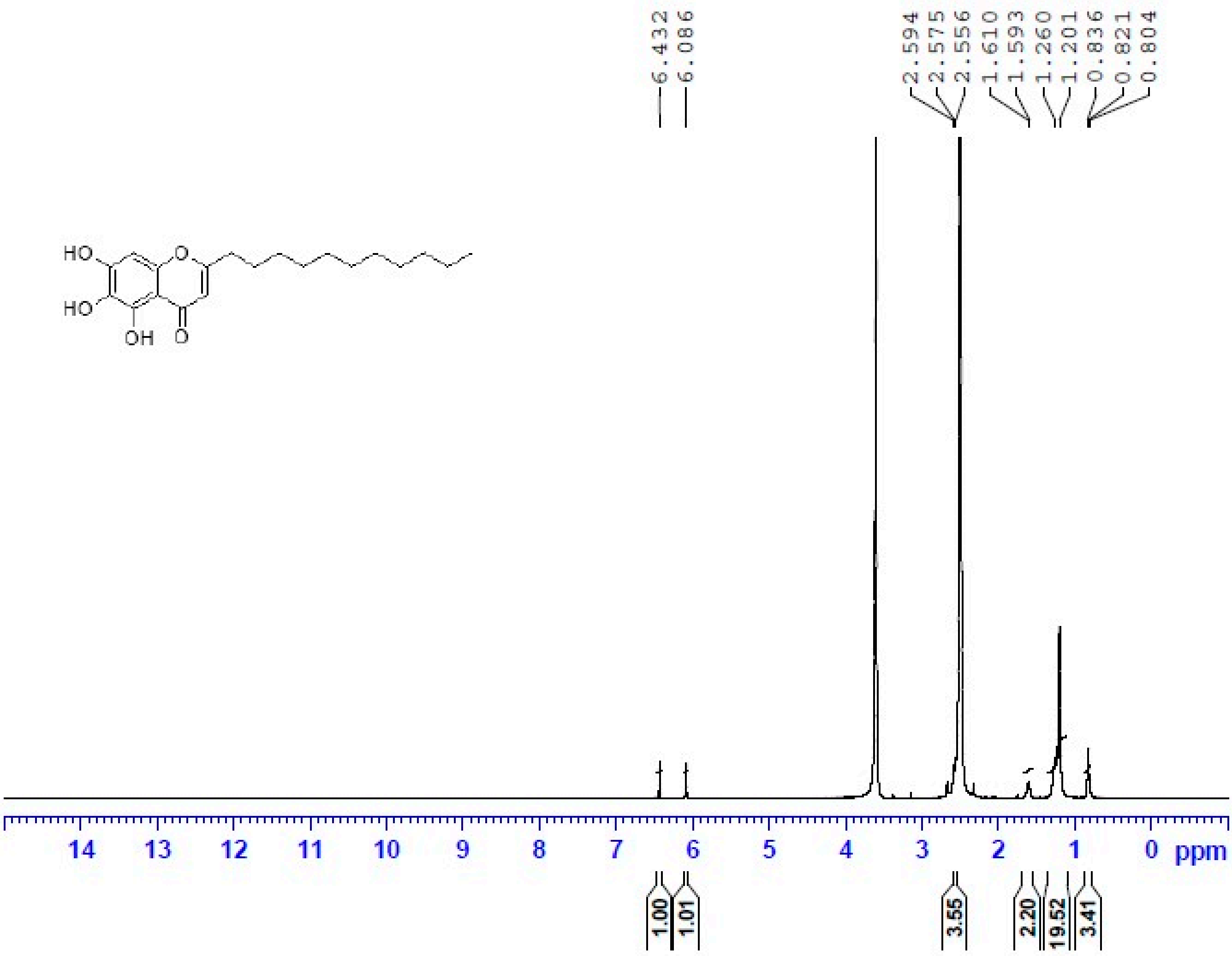

Figure S9 :^{1}H NMR spectra of compound 5 (FNDR-10142)

LC-MS Analysis Report

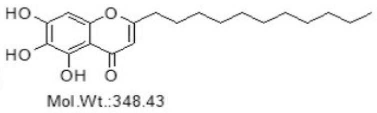

<Sample Information>

10-07-2018_39.lcd

Sample Name	: Demethylation
Sample ID	: ALS18AF094-0041-98-03
Date Acquired	: 7/10/2018 3:20:50 PM
Date Processed	: 7/10/2018 4:58:01 PM
Method File	: LCMS_01.lcm

<LC Chromatogram>

10-07-2018_39.lcd

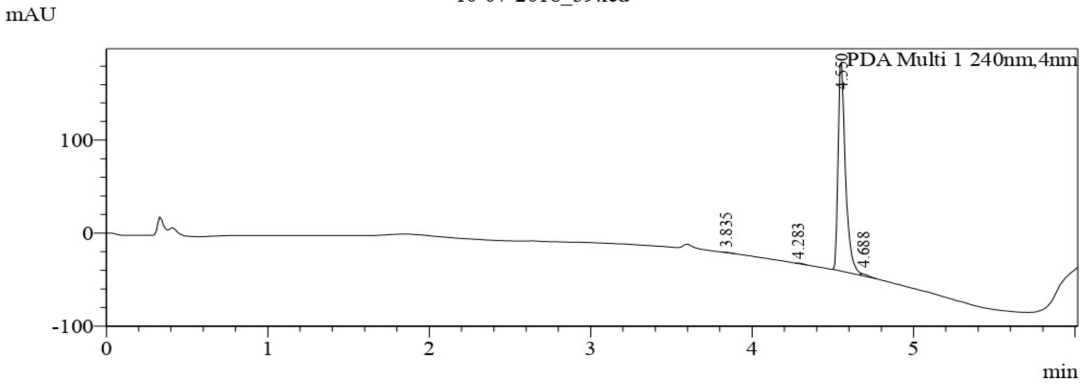

Peak Table 10-07-2018_39.lcd

PDA Ch1 240nm

Peak#	Name	Ret. Time	Area	Area%
1		3.835	372	0.052
2		4.283	726	0.101
3		4.550	711930	99.113
4		4.688	5272	0.734
Total			718299	100.000

<MS Spectrum>

10-07-2018_39.lcd

Line#:1 R.Time:4.633(Scan#:1391)
MassPeaks:8
Spectrum Mode:Single 4.633(1391) Base Peak:349(9281974)
BG Mode:None Segment 1 - Event 1

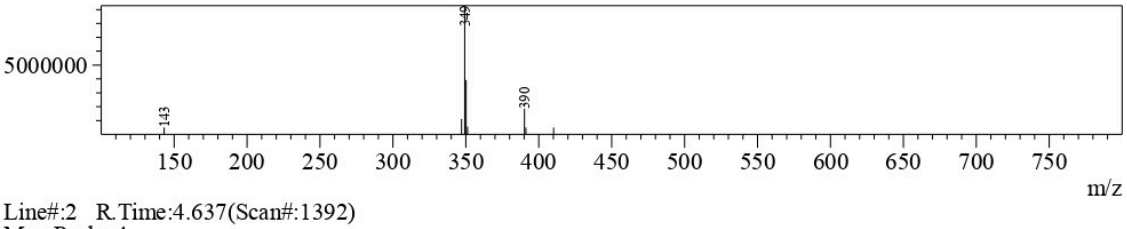

Line#:2 R.Time:4.637(Scan#:1392)
MassPeaks:4
Spectrum Mode:Single 4.637(1392) Base Peak:347(2925844)
BG Mode:None Segment 1 - Event 2

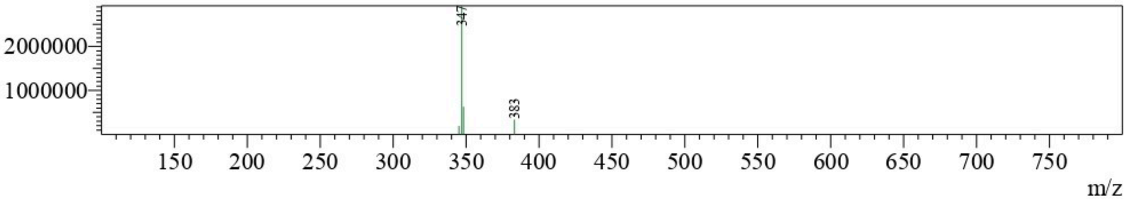

Figure S10 : LC-MS spectra of compound 5 (FNDR-10142)

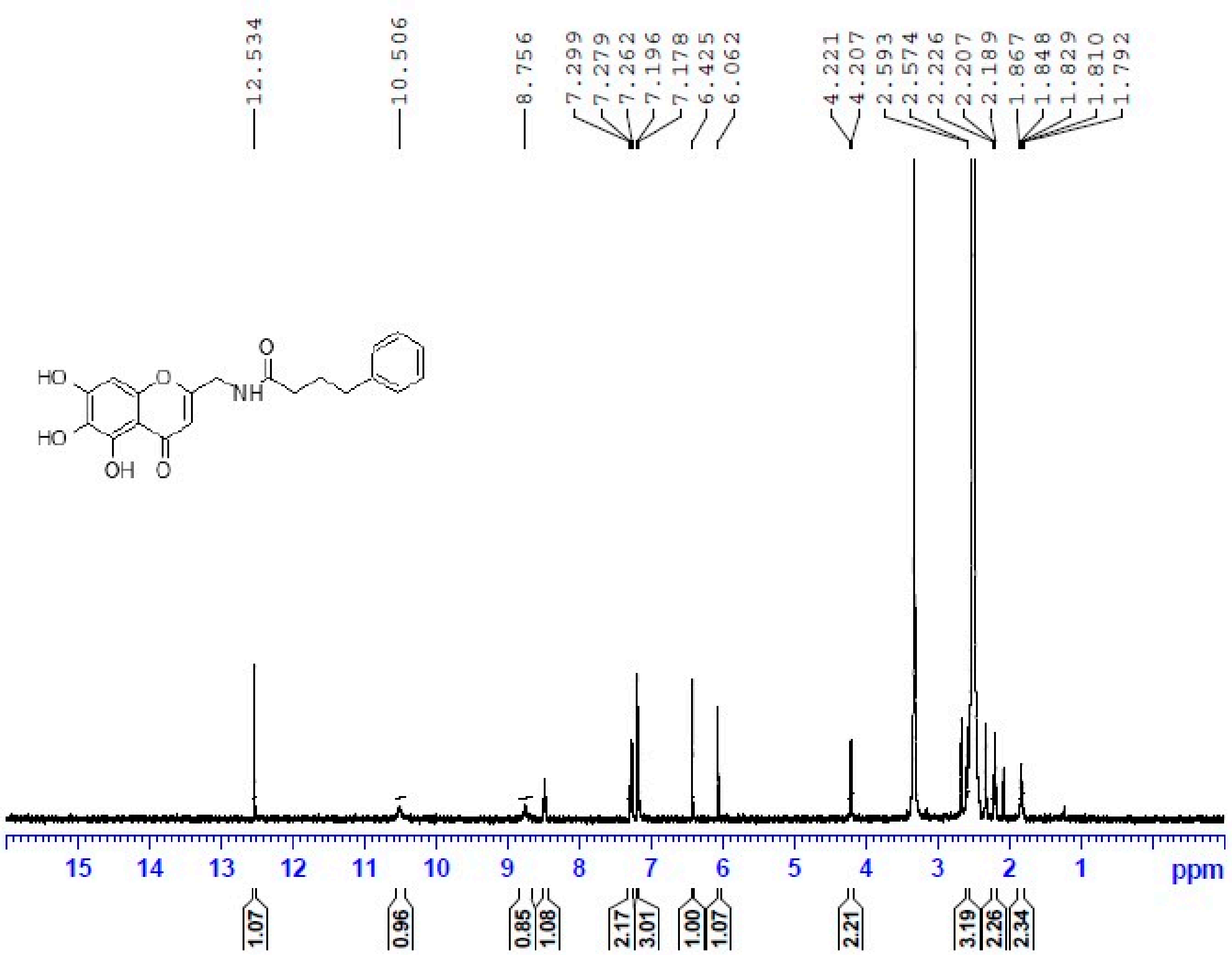

Figure S11: ^{1}H NMR spectra of compound 20 (FNDR-10143)

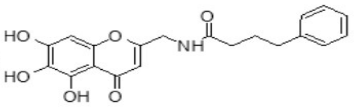

Mol.Wt.:369.36

LC-MS Analysis Report

<Sample Information>

20-07-2018_44.lcd

Sample Name	: Coupling
Sample ID	: ALS18AF094-0056-16-03
Date Acquired	: 7/20/2018 3:43:30 PM
Date Processed	: 7/20/2018 5:36:02 PM
Method File	: LCMS 01.lcm

<LC Chromatogram>

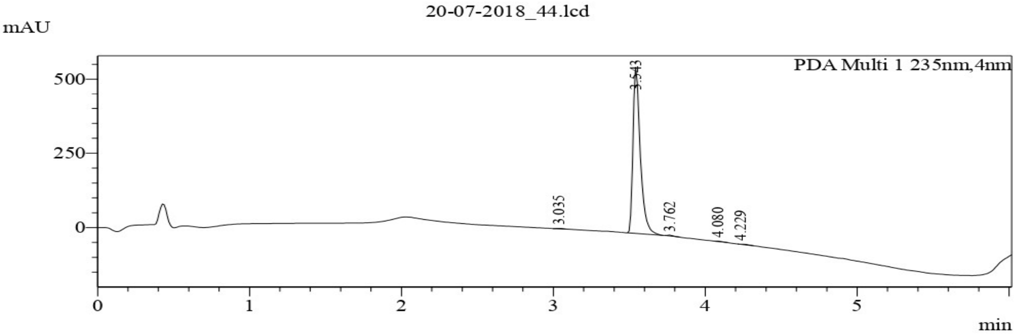

Peak Table 20-07-2018_44.lcd

PDA Ch1 235nm

Peak#	Name	Ret. Time	Area	Area%
1		3.035	3236	0.172
2		3.543	1862435	99.197
3		3.762	6638	0.354
4		4.080	1889	0.101
5		4.229	3318	0.177
Total			1877517	100.000

<MS Chromatogram>

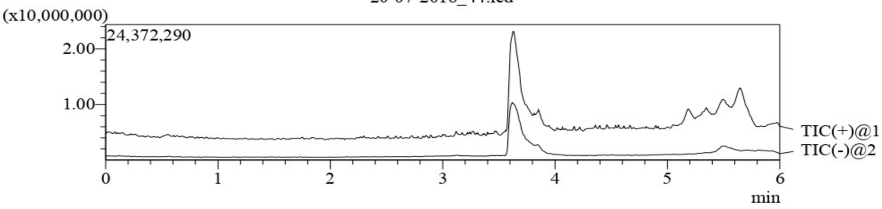

<MS Spectrum>

20-07-2018_44.lcd

Line#:1 R.Time:3.620(Scan#:1087)
MassPeaks:7
Spectrum Mode:Single 3.620(1087) Base Peak:370(8559537)
BG Mode:None Segment 1 - Event 1

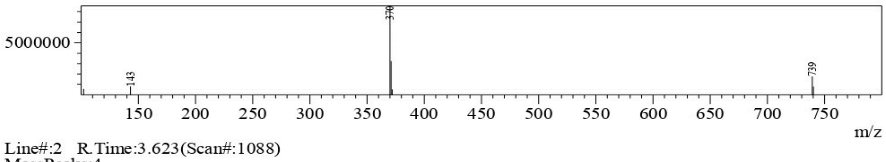

Line#:2 R.Time:3.623(Scan#:1088)
MassPeaks:4
Spectrum Mode:Single 3.623(1088) Base Peak:368(4955544)
BG Mode:None Segment 1 - Event 2

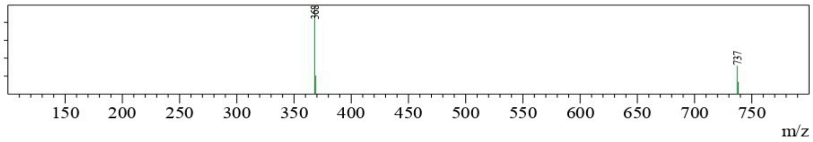

Figure S12: LC-MS spectra of compound 20 (FNDR-10143)

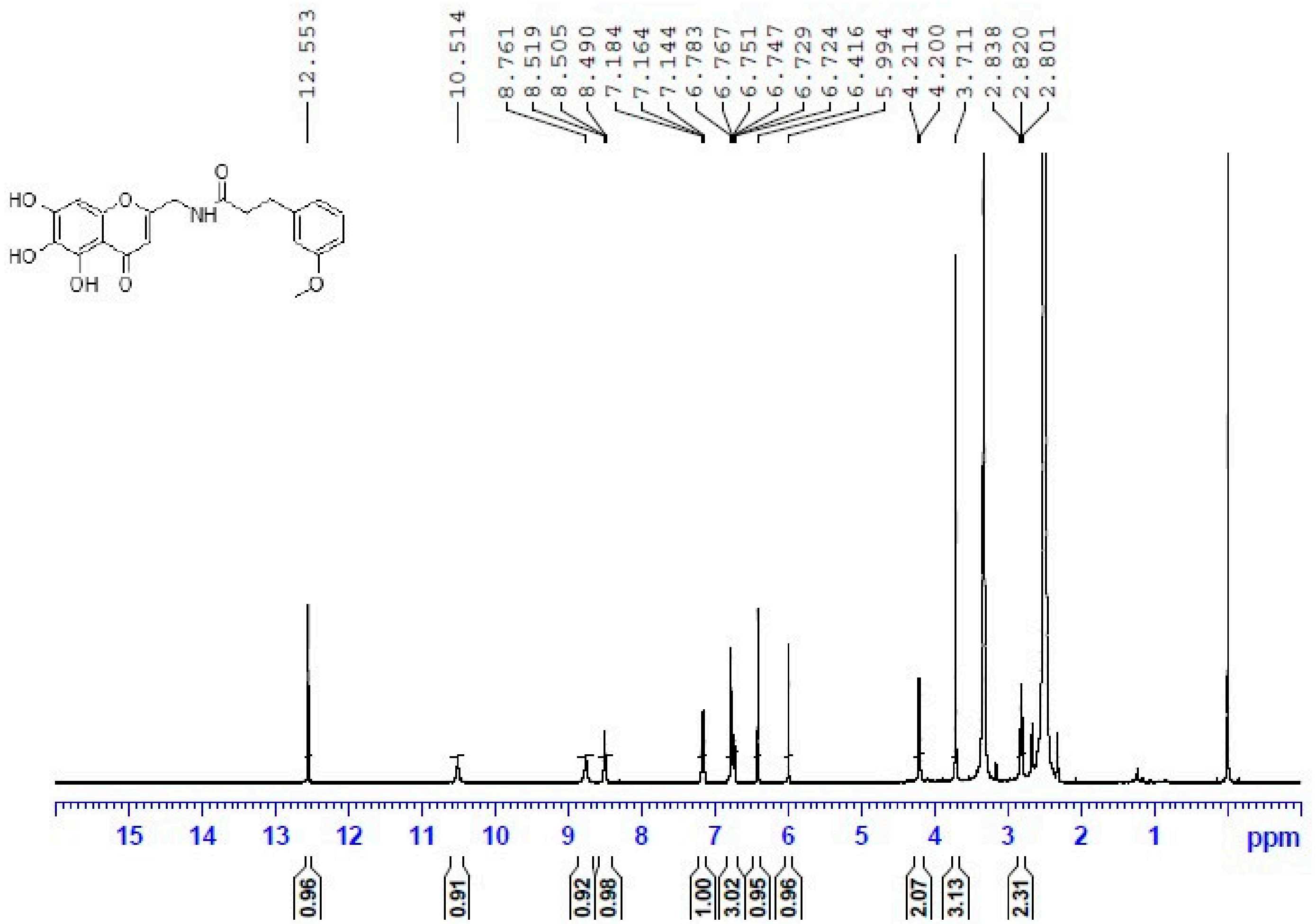

Figure S13 :^{1}H NMR spectra of compound 21 (FNDR-10146)

LC-MS Analysis Report

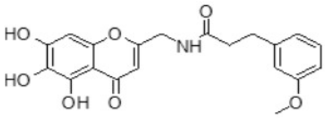

Mol.Wt.:385.36

<Sample Information>

06-08-2018_80.lcd

Sample Name	: Bac-18
Sample ID	: ALS18AF094-0056-34-05
Date Acquired	: 8/6/2018 8:10:16 PM
Date Processed	: 8/7/2018 8:56:29 AM
Method File	: HPLC 01.lcm

<LC Chromatogram>

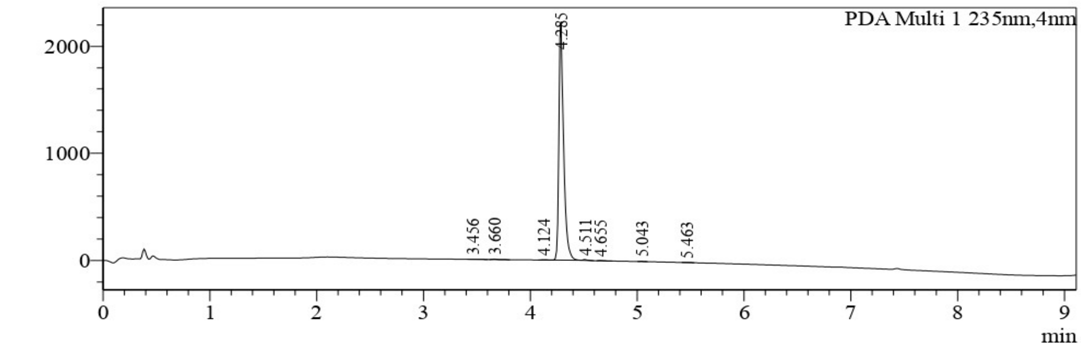

Peak Table 06-08-2018_80.lcd

PDA Ch1 235nm

Peak#	Name	Ret. Time	Area	Area%
1		3.456	4741	0.069
2		3.660	11949	0.174
3		4.124	3953	0.058
4		4.285	6807264	99.106
5		4.511	20147	0.293
6		4.655	7937	0.116
7		5.043	5829	0.085
8		5.463	6875	0.100
Total			6868695	100.000

<MS Spectrum>

06-08-2018_80.lcd

Line#:1 R.Time:4.513(Scan#:1355)
MassPeaks:5
Spectrum Mode:Single 4.513(1355) Base Peak:386(9462178)
BG Mode:None Segment 1 - Event 1

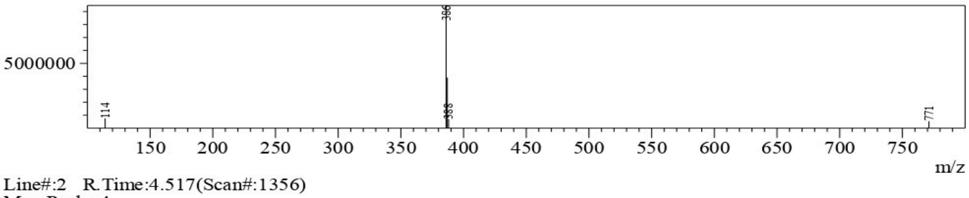

Line#:2 R.Time:4.517(Scan#:1356)
MassPeaks:4
Spectrum Mode:Single 4.517(1356) Base Peak:384(4865388)
BG Mode:None Segment 1 - Event 2

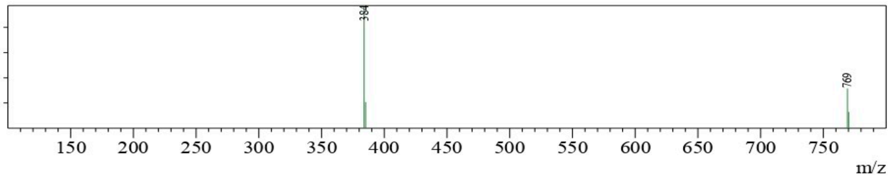

Figure S14 : LC-MS spectra of compound 21 (FNDR-10146)

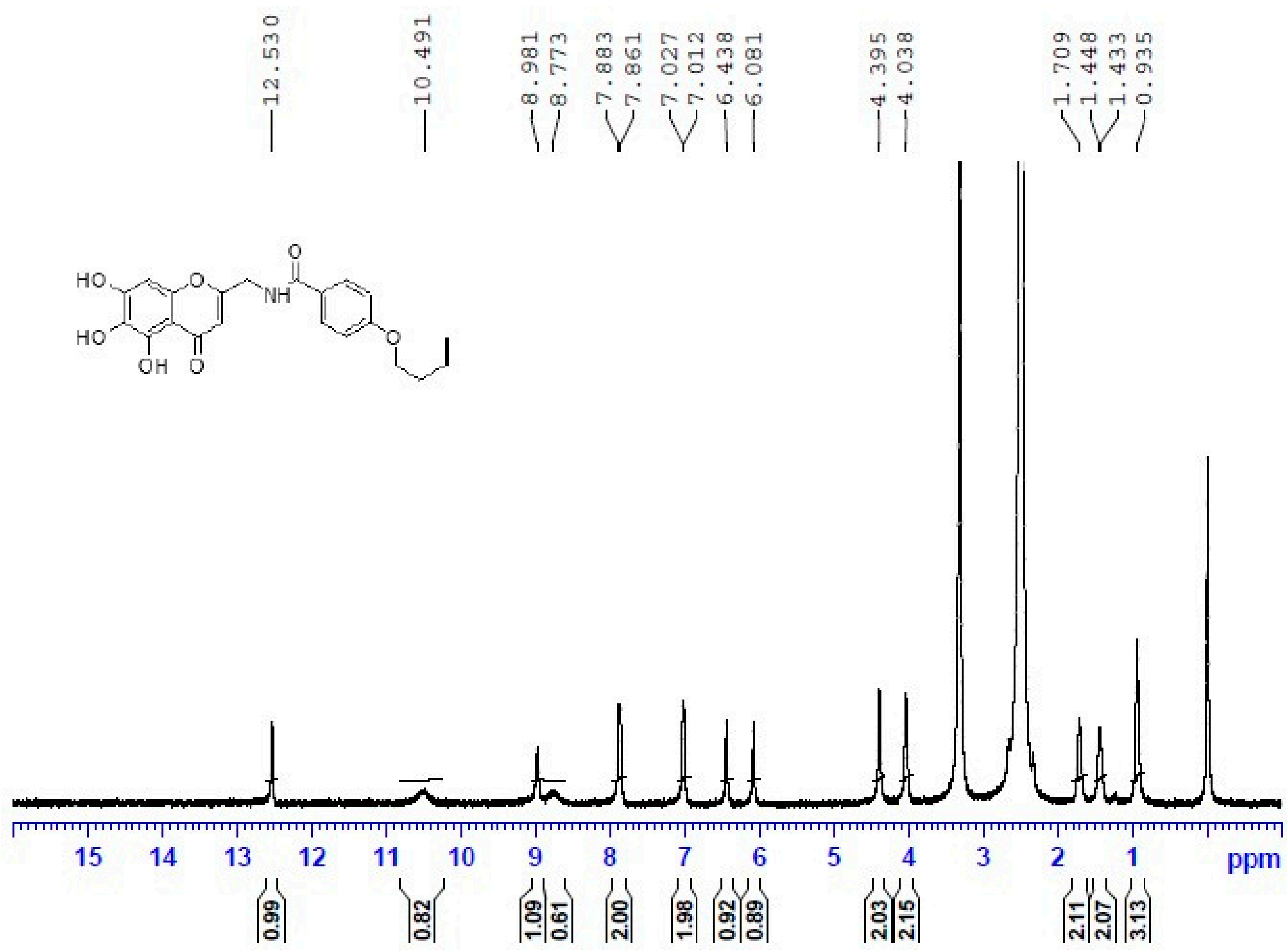

Figure S15: ^{1}H NMR spectra of compound 22 (FNDR-10148)

LC-MS Analysis Report

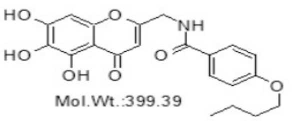

<Sample Information>

13-08-2018_15.lcd

Sample Name	: Bac-21
Sample ID	: ALS18AF094-0055-60-02
Date Acquired	: 8/13/2018 11:03:45 AM
Date Processed	: 8/13/2018 11:29:20 AM
Method File	: LCMS 01.lcm

<LC Chromatogram>

13-08-2018_15.lcd

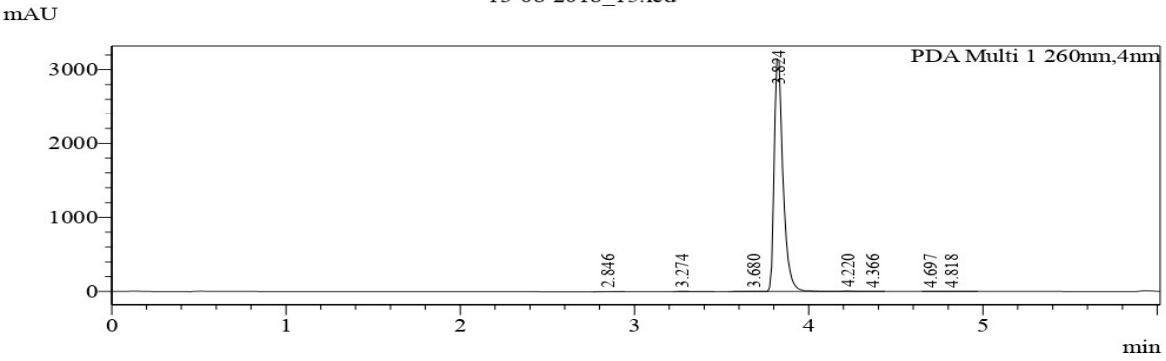

Peak Table 13-08-2018_15.lcd

PDA Ch1 260nm

Peak#	Name	Ret. Time	Area	Area%
1		2.846	8145	0.072
2		3.274	16603	0.148
3		3.680	4348	0.039
4		3.824	11183507	99.431
5		4.220	23202	0.206
6		4.366	1027	0.009
7		4.697	1195	0.011
8		4.818	9431	0.084
Total			11247459	100.000

<MS Spectrum>

13-08-2018_15.lcd

Line#:1 R.Time:3.913(Scan#:1175)
MassPeaks:607
Spectrum Mode:Single 3.913(1175) Base Peak:400(8310720)
BG Mode:None Segment 1 - Event 1

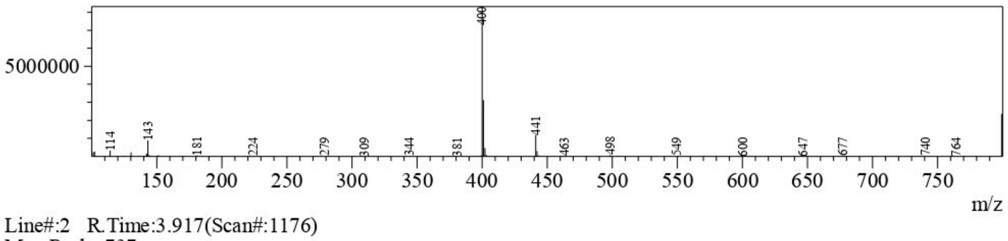

Line#:2 R.Time:3.917(Scan#:1176)
MassPeaks:737
Spectrum Mode:Single 3.917(1176) Base Peak:398(4121336)
BG Mode:None Segment 1 - Event 2

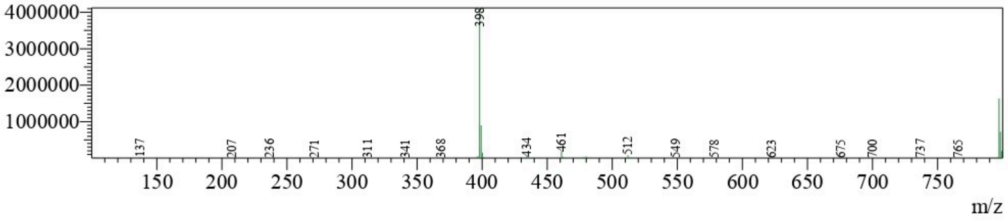

Figure S16: LC-MS spectra of compound 22 (FNDR-10148)

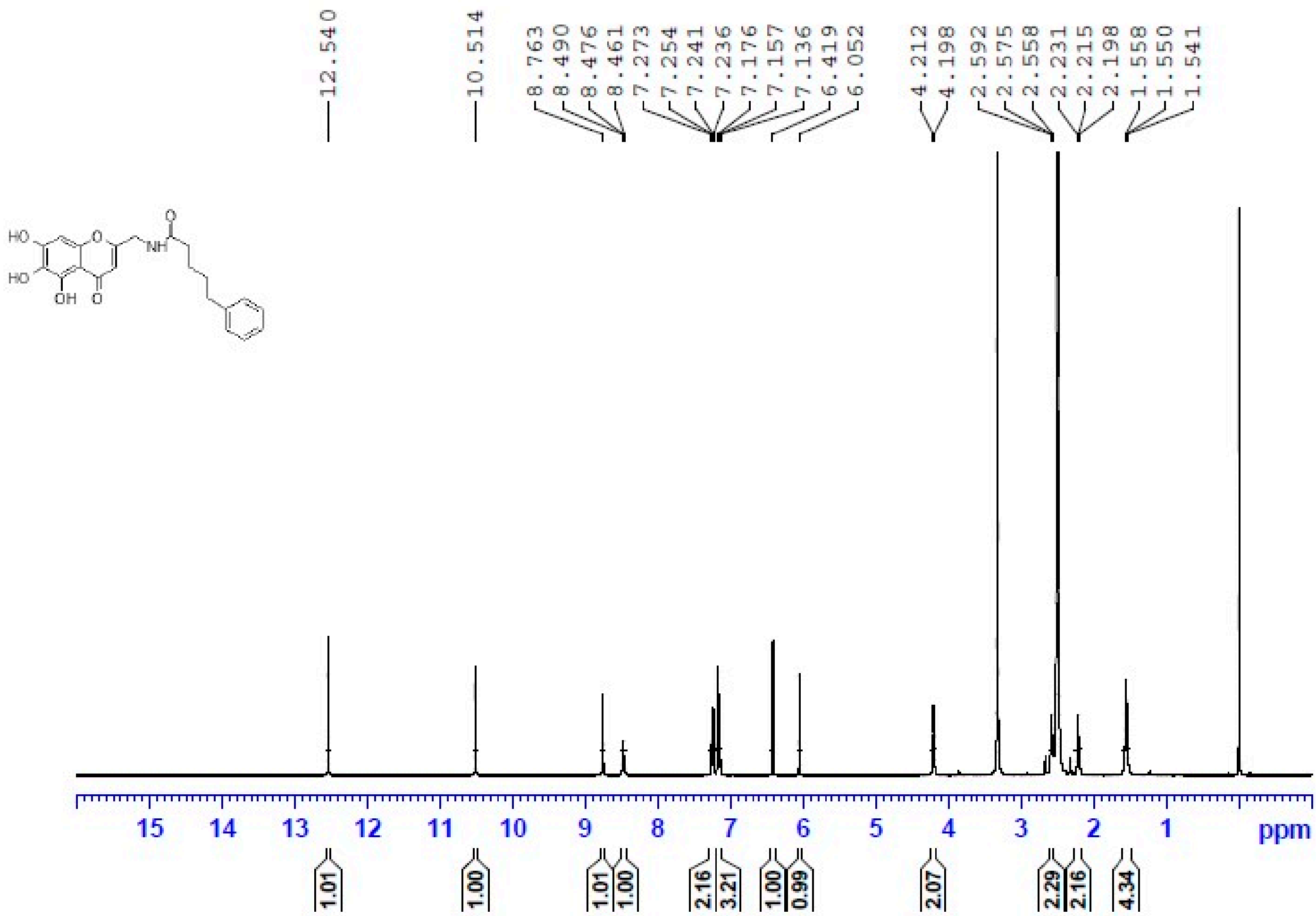

Figure S17:^{1}H NMR spectra of compound 23 (FNDR-10149)

LC-MS Analysis Report

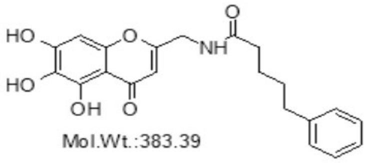

Mol.Wt.:383.39

<Sample Information>

14-08-2018_46.lcd

Sample Name	: Bac 23
Sample ID	: ALS18AF094-0056-49-02
Date Acquired	: 8/14/2018 5:17:45 PM
Date Processed	: 8/14/2018 5:52:37 PM
Method File	: LCMS 01.lcm

<LC Chromatogram>

14-08-2018_46.lcd

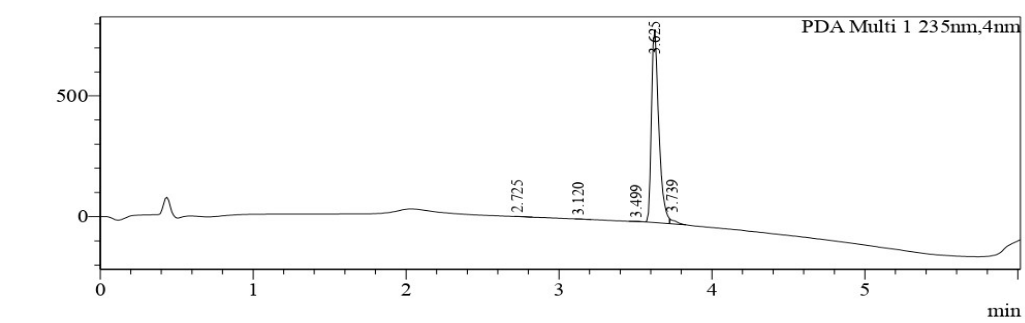

Peak Table 14-08-2018_46.lcd

PDA Ch1 235nm

Peak#	Name	Ret. Time	Area	Area%
1		2.725	741	0.027
2		3.120	1001	0.037
3		3.499	2943	0.108
4		3.625	2671709	98.188
5		3.739	44608	1.639
Total			2721001	100.000

<MS Spectrum>

14-08-2018_46.lcd

Line#:1 R.Time:3.720(Scan#:1117)
MassPeaks:5
Spectrum Mode:Single 3.720(1117) Base Peak:384(8846815)
BG Mode:None Segment 1 - Event 1

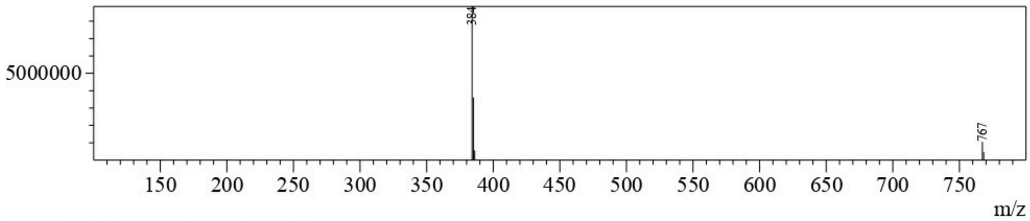

Line#:2 R.Time:3.723(Scan#:1118)
MassPeaks:5
Spectrum Mode:Single 3.723(1118) Base Peak:382(4258883)
BG Mode:None Segment 1 - Event 2

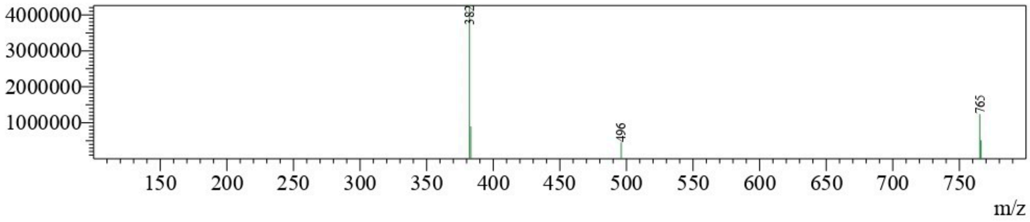

Figure S18: LC-MSspectra of compound 23 (FNDR-10149)

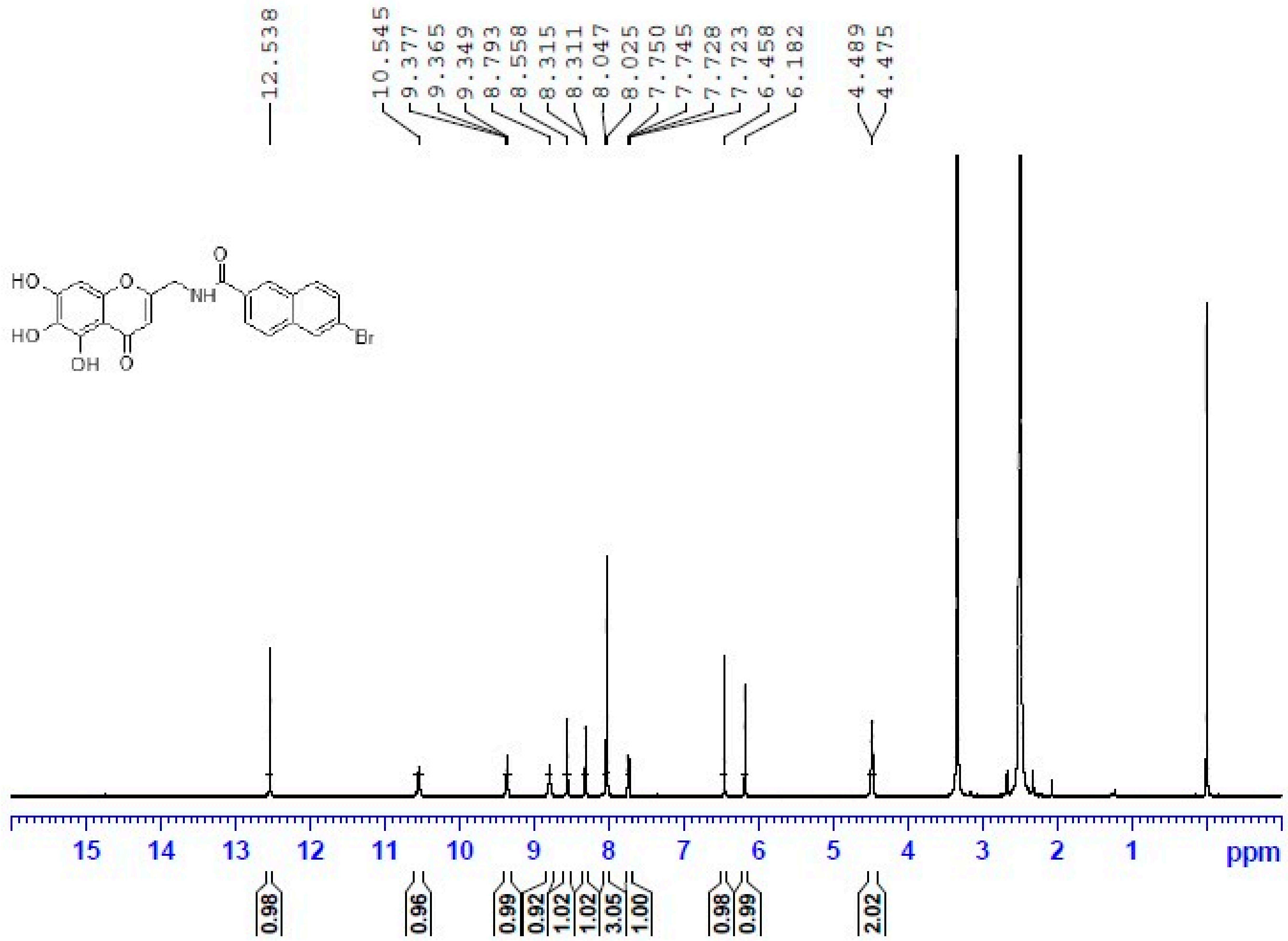

Figure S19: ^{1}H NMR spectra of compound 24 (FNDR-10150)

LC-MS Analysis Report

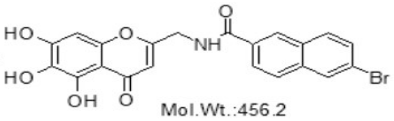

<Sample Information>

17-08-2018_41.lcd

Sample Name	: Bac-24
Sample ID	: ALS18AF094-0056-52-02
Date Acquired	: 8/17/2018 5:30:46 PM
Date Processed	: 8/17/2018 5:42:23 PM
Method File	: LCMS 01.lcm

<LC Chromatogram>

17-08-2018_41.lcd

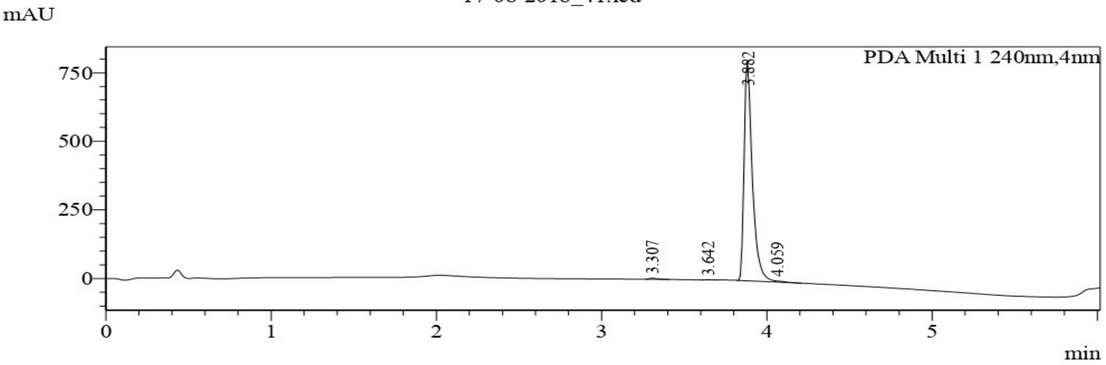

Peak Table 17-08-2018_41.lcd

PDA Ch1 240nm

Peak#	Name	Ret. Time	Area	Area%
1		3.307	15086	0.540
2		3.642	3192	0.114
3		3.882	2762159	98.867
4		4.059	13372	0.479
Total			2793810	100.000

<MS Spectrum>

17-08-2018_41.lcd

Line#:1 R.Time:3.960(Scan#:1189)
MassPeaks:5
Spectrum Mode:Single 3.960(1189) Base Peak:100(1789936)
BG Mode:Averaged 2.880-3.560(865-1069) Segment 1 - Event 1

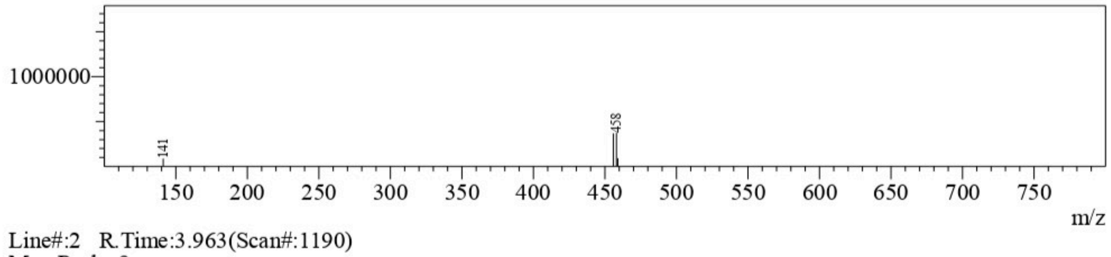

Line#:2 R.Time:3.963(Scan#:1190)
MassPeaks:9
Spectrum Mode:Single 3.963(1190) Base Peak:568(430185)
BG Mode:Averaged 2.883-3.563(866-1070) Segment 1 - Event 2

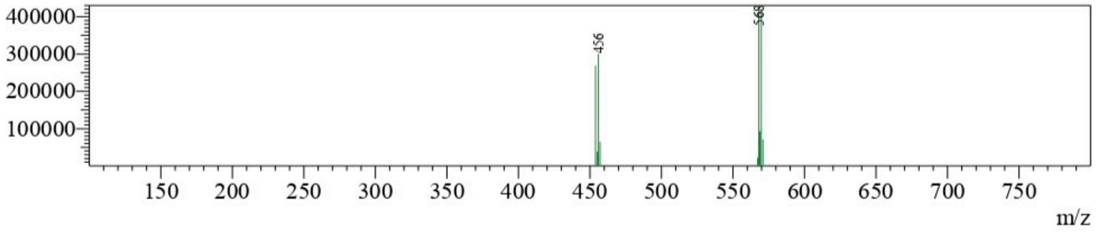

Figure S20: LC-MS spectra of compound 24 (FNDR-10150)
